# Assessing immunogenicity barriers of the HIV-1 envelope trimer

**DOI:** 10.1038/s41541-023-00746-3

**Published:** 2023-09-30

**Authors:** Liridona Maliqi, Nikolas Friedrich, Matthias Glögl, Stefan Schmutz, Daniel Schmidt, Peter Rusert, Merle Schanz, Maryam Zaheri, Chloé Pasin, Cyrille Niklaus, Caio Foulkes, Thomas Reinberg, Birgit Dreier, Irene Abela, David Peterhoff, Alexandra Hauser, Roger D. Kouyos, Huldrych F. Günthard, Marit J. van Gils, Rogier W. Sanders, Ralf Wagner, Andreas Plückthun, Alexandra Trkola

**Affiliations:** 1 Institute of Medical Virology, University of Zurich (UZH), Zurich, Switzerland; 2 Department of Infectious Diseases and Hospital Epidemiology, University Hospital Zurich (USZ), Zurich, Switzerland; 3 Department of Biochemistry, University of Zurich (UZH), Zurich, Switzerland; 4 Institute of Clinical Microbiology and Hygiene, University Hospital, Regensburg, Germany; 5 Institute of Medical Microbiology and Hygiene, Molecular Microbiology (Virology), University of Regensburg, Regensburg, Germany; 6 Department of Medical Microbiology and Infection Prevention, Amsterdam UMC, University of Amsterdam, Amsterdam, the Netherlands; 7 Department of Microbiology and Immunology, Weill Cornell Medical College, Cornell University, New York, USA

**Keywords:** Protein vaccines, Antibodies, HIV infections

## Abstract

Understanding the balance between epitope shielding and accessibility on HIV-1 envelope (Env) trimers is essential to guide immunogen selection for broadly neutralizing antibody (bnAb) based vaccines. To investigate the antigenic space of Env immunogens, we created a strategy based on synthetic, high diversity, Designed Ankyrin Repeat Protein (DARPin) libraries. We show that DARPin Antigenicity Analysis (DANA), a purely in vitro screening tool, has the capability to extrapolate relevant information of antigenic properties of Env immunogens. DANA screens of stabilized, soluble Env trimers revealed that stronger trimer stabilization led to the selection of highly mutated DARPins with length variations and framework mutations mirroring observations made for bnAbs. By mimicking heterotypic prime-boost immunization regimens, DANA may be used to select immunogen combinations that favor the selection of trimer-reactive binders. This positions DANA as a versatile strategy for distilling fundamental antigenic features of immunogens, complementary to preclinical immunogenicity testing.

## Introduction

Elicitation of broadly neutralizing antibodies (bnAbs) that potently block HIV-1 across genetically diverse subtypes is a major goal of HIV vaccine development but has proven extraordinarily challenging^1,2^. Broad and potent neutralization of HIV-1 requires antibody recognition of the native envelope (Env) trimer^3–5^. Most neutralization-sensitive sites are conformationally shielded on the closed prefusion conformation of the trimer, allowing access only of rare antibodies that develop neutralization breadth with time^6^. Multiple factors, including duration of infection, viral load, and viral diversity, contribute to bnAb development during infection^7–11^. To our knowledge, there is currently however no isolated, rapid pathway for the induction of bnAbs that could easily be mimicked in vaccine development. bnAbs develop gradually over time on the basis of a long, complex co-evolutionary feedback loop between the virus and antibody development^12–14^. A wide number of immune system components, including CD4 + T cells, particularly T follicular helper (Tfh) cells, and diverse soluble factors, contribute to bnAb development, but the key regulators that facilitate bnAb induction by vaccination remain to be determined^15^. Hallmarks of HIV-1 bnAbs are their unique features, which frequently include an extreme number of somatic hypermutations (SHM), high cross-reactivity with self-antigens, unusual framework mutations and/or particularly long complementary determining regions (CDRs)^16–22^. Several of these properties can be recognized as abnormal, and cells carrying B cell receptors (BCR) with such features often do not survive, further explaining why the development of bnAbs is rare and requires prolonged time^20,23,24^. Collectively, this accumulation of abnormal traits reflects the difficulty the human immune system has to recognize immunogenic sites on the HIV-1 Env trimer that allow the development of potent neutralizing responses.

Only a fraction of the Env proteins exist as intact trimer on virus and infected cells. Partially or fully dissociated trimers, entry intermediates of Env, and Env precursors released from dying infected cells, however, are present in abundance^25^. Highly immunogenic regions on these Env derivatives give rise only to strain-specific, non- or weak neutralizing antibodies^26^. The strong immune-focusing on such non-neutralizing epitopes is considered detrimental in the context of vaccines since competition in germinal center reactions by abundant, high-affinity non-neutralizing responses limits the chances for bnAb evolution^15,27–31^. A prominent immunodominant region is the V3 loop within gp120^32,33^. Consistent with its critical function in entry through interaction with the coreceptor, the V3 loop is hidden inside the native prefusion conformation of the Env trimer and becomes accessible only during the entry process after Env binding to the CD4 receptor^34^. Antibodies against the V3 loop are produced in large numbers in HIV-1 infection but are essentially non-neutralizing because they cannot access V3 on the prefusion trimer^35–37^. Consequently, current approaches to bnAb-inducing immunogens attempt to eliminate V3 reactivity either by generating highly stabilized soluble Env trimers that fully protect the V3 loop or by generating epitope-specific subunit immunogens that lack V3^38–43^. However, while these stabilized trimers successfully limit undesired antibody responses to V3 and other regions on the open trimer, the induction of bnAb responses by them remains sporadic^1^.

Herein, we introduce the designed ankyrin repeat protein (DARPin)^44^ technology as an in vitro tool to assess the immunoreactivity of the closed Env trimer. The DARPin technology was originally developed to provide alternatives to antibodies and a way to select high-affinity binding proteins from high-diversity libraries (theoretical diversity of 5.2 × 10^15^). Although the small DARPin proteins are structurally different from antibodies and have a different binding mode, they equal their capacity for high affinity binding^45,46^. We recently showed that, similar to the human antibody response to HIV-1, high numbers of V3-reactive binders are selected from DARPin libraries panned against HIV-1 Env proteins^37^. Importantly, among the V3-reactive binders we also selected some broadly neutralizing DARPins (bnDs), demonstrating that DARPins do share the capacity for highly efficacious inhibition of HIV-1 with bnAbs^37^. Building on this, here we perform **D**ARPin **An**tigenicity **A**nalysis (DANA) to investigate the antigenic space of highly stabilized Env trimers in the context of different strategies to reduce V3 immunodominance. The overall aim of our study was to create through DANA a purely in vitro screening tool with means to extrapolate information on Env immunogens used in prime-boost vaccination regimens.

## Results

### In vitro assessment of Env trimer antigenicity using the DARP in technology

**Fig. 1:**
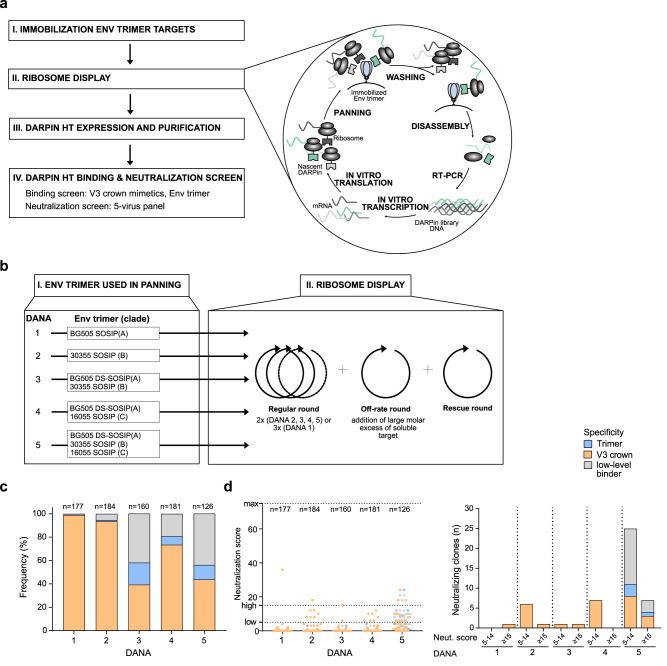
DANA in vitro assessment of Env trimer antigenicity. **a** Workflow of DANA. **b** DANA 1–5: Overview of recombinant HIV-1 Env trimer used for panning (I) and type and number of ribosome display selection rounds (II). **c** and **d** DARPin clones with valid ORF derived from the randomly picked 190 clones in each DANA screen were analyzed for binding and neutralizing properties (*n* = 126–184). Data are derived from single experiments. **c** Binding properties of DARPins obtained from DANA 1–5, based on ELISA, using Env trimer probes and the V3-crown mimetic peptide V3-IF (BG505) as specified in Supplementary Table 3. Trimer binding is categorized as binding to at least one of several trimers but not the V3-mimetic. V3 specificity includes solely V3-reactive and V3- and trimer dual-reactive clones. Low-level binders categorize DARPins with no trimer- and no V3-reactivity >3-fold over background. **d** Neutralization capacities of DARPins derived in DANA 1–5 against a multi-clade 5-pseudovirus panel according to a neutralization score reflecting breadth and potency. The max. score is 75, a score 5–14 indicates low neutralizing activity, a score of >15 indicates high neutralizing activity. The left panel depicts the neutralization score, the right panel the number of clones with low and high neutralizing activity, respectively.

Isolation of target-specific DARPins from original high-content DARPin libraries is achieved by ribosome display after several rounds of panning and binder enrichment^47,48^ (Fig. 1a, b, Supplementary Fig. 1). We reasoned that this experimental design with successive rounds of target-panning and selection represents a controlled setting to study accessibility of antigenic sites on stabilized Env trimer immunogens. Consecutive rounds of selection can be conducted with a single type of target protein, mimicking homotypic immunization (a single Env immunogen for prime and boost) or with different targets, reflecting heterotypic prime-boost strategies utilizing diverse Env immunogens. Resulting DARPin pools can be screened for the presence of target-specific DARPins that contain features of interest; in the present study these were Env- and epitope specificity and virus neutralization capacity (Fig. 1a). The quantity of Env- and epitope-specific DARPins together with their genetic diversity as well as neutralization breadth and potency selected by a given antigen can be assessed, providing an in vitro surrogate measure of its antigenicity and the relative dominance of certain epitopes over others.

We refer to this test as **D**ARPin **An**tigenicity **A**nalysis (DANA) and probed its potential using four candidate Env-trimer immunogens as panning targets in five independent DARPin screens (DANA 1–5) with either homotypic or heterotypic target composition (Fig. 1b, Supplementary Table 1, Supplementary Table 2). The Env immunogens were stabilized as soluble trimers using the original SOSIP^3^ or DS-SOSIP^49^ design and included Envs that have undergone extensive pre-clinical and clinical testing as well as experimental Env trimers (Supplementary Table 1). The included trimers covered diverse HIV-1 subtypes, bound known bnAbs as expected, and represented fully closed to partially open immunogens based on reactivity with the CD4-binding site-specific antibody b12 and the co-receptor-mimicking antibody 17b that bind open Env conformations^50^ (Fig. 1b, Supplementary Fig. 2, Supplementary Fig. 3, Supplementary Table 2). A modest V3 exposure on stabilized Env trimers is common^3,42,49^ and was also detected for most trimers included (Supplementary Fig. 2).

The design of the DANA screens included 4–5 individual DARPin selection rounds with an optional off-rate step before the final round (Fig. 1b). Randomly picked 190 DARPin clones of each selection were screened and characterized for Env specificity and neutralization capacity (Fig. 1c, d). We focused in the Env specificity assessment on two features: first, reactivity with the Env-trimer, a requirement for most bnAbs, and second, V3-crown reactivity, as an indicator of undesired immunodominant reactivity. V3 binding was assessed using the BG505-derived V3-crown mimetic peptide V3-IF, which reacts well with V3-crown specific DARPins, monoclonal Abs and plasma from people infected with HIV-1^37^. Env specificity was divided into three categories: (i) reactivity with panning target Env-trimer(s) but no reactivity with V3-IF; (ii) reactivity with V3-IF (including clones with and without Env trimer reactivity); (iii) low-level binders (trimer and V3 binding undetectable or <3-fold above background).

We observed a massive dominance of V3-crown-reactive DARPins in the two homotypic DANA screens using clade A BG505 SOSIP (DANA 1) and clade B 30355 SOSIP (DANA 2) as panning targets, where V3-reactive clones accounted for 99% and 94% of the response, respectively (Fig. 1c). Only a minute fraction of clones of 1% were trimer-specific without targeting V3, mirroring what has been widely observed in HIV Env vaccine studies^38,51^. Interestingly, the trimer-binding fraction increased in heterotypic DANAs compared to homotypic DANAs. For example, in a heterotypic DANA screen alternating between BG505 DS-SOSIP and 30355 SOSIP (DANA 3), the V3-reactive clones were substantially reduced (accounting for 39%) and the proportion of trimer-reactive clones was increased to 20%, supporting the need to avoid dominant responses in order to allow for trimer-specific binders to be retained during selections. Of note, there was a parallel increase in the proportion of clones with low-level binding that did not react above threshold (trimer and V3 binding undetectable or >3-fold above background) with V3-IF or the trimers tested in the screening (Fig. 1c, Supplementary Table 3), suggesting that reduced V3 dominance enabled enrichment of DARPins that bound with lower affinity to the targets. A heterotypic DANA regimen combining BG505 DS-SOSIP with clade C 16055 SOSIP (DANA 4), albeit less pronounced, confirmed the beneficial effect of exposure to genetically diverse antigens on increasing trimer reactivity while decreasing V3 responses as compared to DANA 1. The fact that 30355 SOSIP is genetically more divergent from BG505 in V3 than 16055 SOSIP (Supplementary Fig. 3) may have benefitted DANA 3 selection. We explored this further in a triple combination (DANA 5), where the pairing of BG505 DS-SOSIP/16055 SOSIP/30355 SOSIP resulted in frequencies of trimer- and V3-reactive clones comparable to DANA 3, suggesting that the combination with 30355 SOSIP, in particular, had a trimer-focusing effect.

The five DANA screens were then evaluated for neutralization breadth and potency against a 5-virus cross-clade panel and a neutralization score was used to distinguish DARPins with high, low, or no neutralizing activity based on scores ≥15, 5–14, <5, respectively (Fig. 1d, Supplementary Fig. 4, Supplementary Table 3, Supplementary Table 4). Overall, neutralization activity yielded in the five DANA screens was modest. DANA 1 and DANA 3 scored lowest with only 1% and 2% neutralizing clones (Fig. 1d). DANA 2 and DANA 4 achieved a higher fraction of clones with neutralization activity, even though most of them appeared to be not very potent. Most intriguingly, the triple combination of Env trimers in DANA 5 scored best, with overall 25% of DARPins showing measurable neutralization activity. Across all five screens, mostly V3-reactive clones harbored neutralization activity (Fig. 1d). The exception was DANA 5, where trimer-reactive and low-level binders outweighed the V3-directed clones.

### High DARPin mutation frequency in DANA reveals antigen complexity

**Fig. 2:**
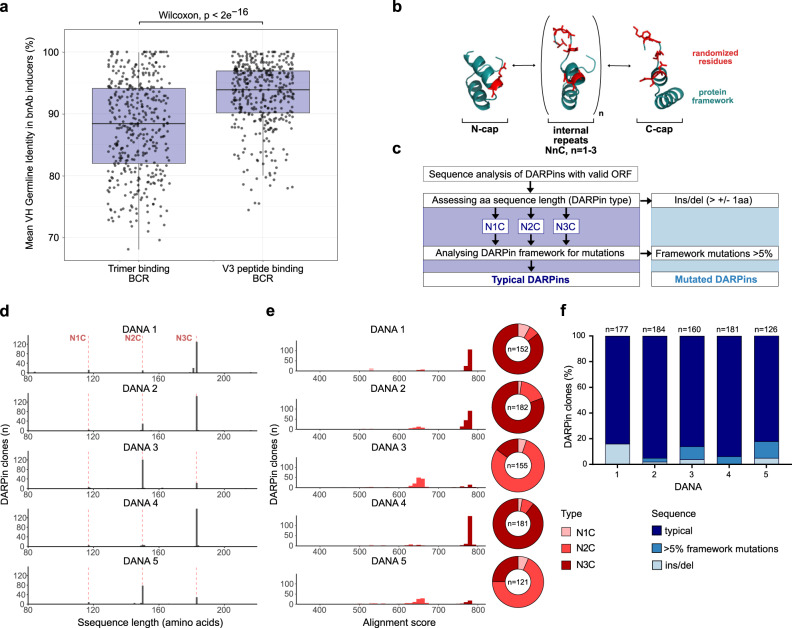
High DARPin mutation frequency in DANA is correlated to antigen complexity. **a** Comparison of mean VH germline identity (GLI) of trimer- (*n* = 444) and V3 peptide-reactive (*n* = 431) clonotypes among 80,963 B cell receptor (BCR) sequences from 19 HIV-1 bnAb inducers (total 21 PBMC samples) analyzed by Libra-seq. Trimer- and V3-binding double-positive BCRs were excluded. Boxplots indicate median (middle line), upper and lower quartiles (box limits) and 1.5× interquartile ranges (whiskers). Mean VH GLIs were compared by two-sided Wilcoxon rank-sum test. **b** Generic DARPin design comprising amino (N-cap) - and carboxyterminal (C-cap) ankyrin repeats, flanking up to three internal ankyrin repeats. Conserved framework in cyan, positions randomized in the library in red. **c** Sequence analysis workflow. DARPins with valid ORF were categorized into N1C, N2C or N3C (117, 150, and 183 amino acids respectively)-type DARPins. DARPins with insertions/deletions ( > 1 aa) were considered mutated. DARPins were pairwise aligned to respective consensus sequences of the same DARPin type (N1C, N2C, N3C) and alignment scores calculated. DARPins with >5% framework substitutions were considered mutated. **d** DARPin length distribution for DANA 1–5. Red dotted lines indicate length for typical N1C, N2C and N3C DARPins. **e** DARPin framework mutation analysis by alignment scores and DARPin type distribution for DANA 1–5. Higher alignment scores indicate higher similarity to the DARPin-type consensus sequence. DARPins with insertions/deletions ( > 1aa) are not considered in this analysis. **f** DARPin frequency without mutations (i.e., typical DARPins), with insertions/deletions and with >5% framework mutations. All DARPins with valid ORF (*n* = 126–184) were analyzed.

Trimer-specific bnAbs often target epitopes with limited access, involving glycan and protein components^52,53^. Due to this complexity, they require longer development times to allow for more extensive SHM and achieve high binding affinity^13,14,54,55^. To gain insight into the difference in SHM of antibodies depending on their epitope accessibility, we examined antibody repertoires in a group of 19 bnAb inducers identified in the Swiss 4.5 K bnAb screen^7,11^. Using stabilized Env trimers and V3 loop peptides as models of immunogens with low and high epitope accessibility, respectively, we compared single cell B cell receptor (BCR) sequences from memory B cells specifically mapped to either SOSIP-stabilized Env trimers or V3 loop antigens with Libra-Seq^56^ (Fig. 2a). The blood specimen from bnAb inducers included in this analysis were sampled after 1–17 years of infection. Low and high access antigen responses had equal chances to mature, allowing direct comparison of SHM values. BCRs in these bnAb inducers that were reactive with one of the three tested stabilized Env trimers showed significantly lower median germline identity (GLI) than BCRs reactive with the V3 loop isolated from the same time point (Fig. 2a and Supplementary Fig. 5), illustrating that not prolonged exposure to the antigen per se but disparate evolution pathways account for the higher SHM observed in trimer specific bnAbs.

We therefore considered the possibility that DARPins, although using a completely different protein architecture, may be influenced by similar antigenic properties, and therefore DARPin mutation frequencies in DANA screens may likewise provide insight into epitope accessibility on the studied antigens. The consensus design of DARPins consists of N-terminal and C-terminal capping repeats (N-cap, C-cap) and internal ankyrin repeats that have conserved framework regions and randomized positions^44,46^ (Fig. 2b and Supplementary Fig. 1). Many bnAbs gain access to difficult-to-reach epitopes through long CDRH3 regions^53,57,58^. Owing to their design, standard DARPins lack the option of gaining access to constrained regions by elongating loops as the synthetic library used here does not encode them^44^, even though such constructs have been made^59^. Typically, the synthetic DARPin libraries will not contain short insertions and deletions nor loss of individual repeats, but occasionally such clones derived by PCR errors are amplified when no other solution to high-affinity binding may exist, e.g., by steric restriction^60,61^.

For DANA, we utilized an N3C DARPin library that contained three internal repeats flanked by N- and C-caps. A loss of internal repeats would therefore be reflected by the enrichment of N1C or N2C DARPins instead of expected N3C clones of the original library. Another way to gain access to the trimer could involve rare mutations in the conserved framework region of the scaffold to alter the overall DARPin structure^61^. This could lead to non-specific binding in some cases, e.g., via hydrophobic surfaces that are exposed by partial unfolding. To assess if and which of these modifications occurred, we assessed the following sequence parameters: (i) definition of DARPin-type based on the number of internal repeats (N1C, N2C, N3C) and DARPins with small insertions or deletions and (ii) DARPin framework mutations (Fig. 2c). To assess mutation frequencies in the DARPin framework (i.e., not considering the randomized positions), we established an alignment score that records the similarity to defined DARPin reference sequences in analogy to GLI identity recorded for BCRs (Supplementary Fig. 6c) in addition to listing position-specific mutations (Supplementary Fig. 7).

For DANA 1, we detected a total of 84% of clones with typical DARPin features (i.e., all DARPins that are not categorized as mutated outside the randomized regions) (Fig. 2c, f), the majority of which were of the N3C type as expected from the starting library (Fig. 2e) and had a high identity in DARPin framework alignment to a reference sequence (Fig. 2e, f). Mutation analysis of DANA 2–5 revealed that the frequency of framework-mutated clones increased in DANA 3 and DANA 5, the two immunogen regimens that exhibited the highest proportion of trimer-reactive clones, concomitant with the lowest V3 dominance (Figs. 1c, 2e, f). Analyzing the frequency of the different mutation categories, we saw strong enrichment for smaller N2C DARPins in DANA 3 and DANA 5 using clade B 30355 SOSIP and clade A BG505 DS-SOSIP, but not in the other three screens, suggesting that this particular combination favored their selection (Fig. 2e). Smaller N2C DARPins show increased neutralization abilities in particular for DANA 5. Interestingly, trimer-specific binders from DANA 3 and 5 in particular acquired sequence alterations (Fig. 2f, Fig. 5, Supplementary Fig. 8). Particularly notable, the triple heterotypic DANA 5 yielded more neutralizing DARPins compared to DANA 3 and 4. In conclusion, trimers based on the first generation SOSIP design alone selected mainly V3-specific DARPins with low neutralizing capacity, similar to the predominant V3 response observed in vaccine studies^38,51^. In contrast, the use of two or three trimers with genetically divergent V3 in alternate rounds resulted in an amplification of clones that bound the trimer but not V3. This effect was modest when combining clade A and C trimers (DANA 4), but more pronounced in combinations with a clade B trimer featuring a comparatively divergent V3 (DANA 3 and 5, and Supplementary Fig. 3). Interestingly, the trimer-binding clones frequently carried framework mutations reminiscent of the high level of somatic hypermutation observed with trimer-binding antibodies.

### Reduction of V3 dominance increases selection of trimer-reactive clones

**Fig. 3:**
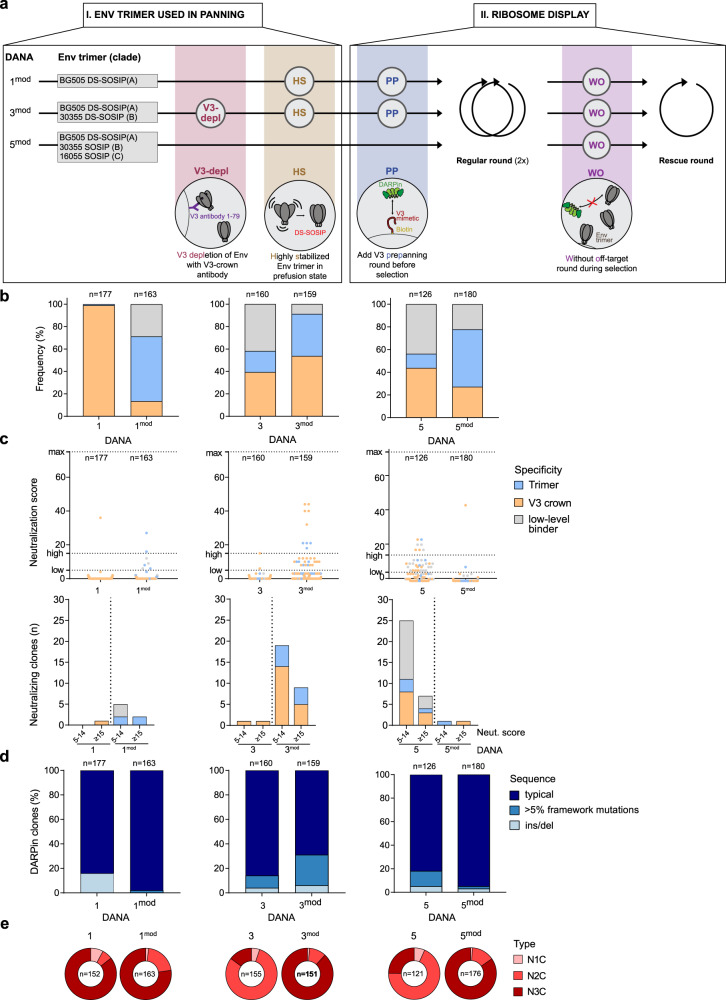
Reduction of V3 dominance increases selection of trimer-reactive clones. **a** Modifications in DANA 1^mod^, 3^mod^, 5^mod^ focus on (I) recombinant HIV-1 Env trimer panning targets and (II) type and number of ribosome display selection rounds. V3-depl: **depl**etion of open, V3-exposing trimers from panning trimer preparation through V3 mAb binding); HS: use of **h**ighly **s**tabilized DS-SOSIP trimer; PP: **p**re-**p**anning of DARPin library with V3 peptide to remove V3-reactive DARPins before DANA; WO: DANA **w**ithout **o**ff-rate round that would favor selection of high-affinity binders. **b**–**e** Comparison of binding, neutralizing and sequence properties of DARPins with valid ORF (*n* = 159–163) among 190 randomly picked clones from each DANA. Data are derived from single experiments. **b** Binding properties of DARPins based on ELISA using Env trimer probes and the V3-crown mimetic peptide V3-IF (BG505) (see Table S4). Trimer binding is categorized as binding to at least one of several trimers but not the V3-mimetic. V3 specificity includes solely V3-reactive and V3- and trimer dual-reactive clones. Low-level binders categorize DARPins with no trimer- and V3-reactivity >3-fold over background. **c** Neutralization score against a multi-clade 5-pseudovirus panel reflecting breadth and potency. The max. score is 75, a score 5–14 indicates low, a score of >15 high neutralizing activity. The left panel depicts the neutralization score, the right panel the number of clones with low and high neutralizing activity, respectively. **d** DARPin frequency without mutations (i.e., typical DARPins), with insertions/deletions and with >5% framework mutations. **e** Distribution of DARPin types.

The necessity to reduce V3 reactivity in HIV immunogen design has long been realized^15,38,39,62^. Taking advantage of the highly adaptable DARPin system, we probed a series of measures to decrease V3 responses and increase trimer and neutralization activity in DANA (Fig. 3a). In a first approach, we explored the impact of the off-rate selection step. While enrichment of high-affinity binders will be key for potent neutralization, the off-rate selection may disproportionally favor high-affinity V3 responses over less affine trimer-reactive clones. The latter may have difficulties in achieving higher affinity due to the dense covering of the trimer protein surface with glycans^63^, or less ideal paratope shape complementarity, resulting in the survival of immunodominant V3-crown specific clones, as observed in germinal center reactions^27,28^. We explored this in DANA 5 modified (DANA 5^mod^) that lacked the off-rate step but was otherwise identical to DANA 5.

Indeed, without the off-rate step, DANA 5^mod^ showed an impressive increase in trimer reactivity paired with lower V3 dominance and lower mutation frequency (Fig. 3b, Fig. 3d). Neutralization activity that was notably high in DANA 5, could however not be recapitulated, underlining the importance of affinity maturation to reach potency (Fig. 2c). Nonetheless, due to the fact that trimer reactivity was increased, we rated the removal of the off-rate step as a potentially beneficial protocol modification and combined it with additional measures in the next exploratory screens DANA 1^mod^ and DANA 3^mod^, which built on DANA 1 and DANA 3, respectively (Fig. 3a). Two further changes were introduced in DANA 1^mod^: the BG505 SOSIP trimer in DANA 1 was replaced by a DS-SOSIP version, owing to its higher conformational stabilization, resulting in higher efficacy in shielding CD4-induced epitopes including V3^49^. To decrease the potential impact of V3 dominance further, we pre-panned the starting DARPin library against V3-crown peptides to remove highly V3-reactive clones from the library. The three combined measures, library V3 pre-panning, use of DS-SOSIP and absence of off-rate selection, resulted for DANA 1^mod^ in a marked increase of trimer reactivity with a small increase in neutralization activity compared to DANA 1 (Fig. 3b–d).

To build on this observation, for DANA 3^mod^ we kept the three measures used in DANA 1^mod^ and in addition used Env preparations for panning from which trimers exposing V3 were partially removed (Supplementary Fig. 9). These combined measures had an impressive effect on DANA 3^mod^ which, compared to the original DANA 3, hugely increased the frequency of neutralizing trimer-directed and V3-crown directed clones (Fig. 3c). The increase in trimer-binding reactivity was paired with a slightly higher mutation frequency (Fig. 3d). However, despite reducing V3 exposure in the panning Env trimers, V3-crown targeting binders remained at a high level. Notably, a significant proportion of trimer-reactive clones harbored mutations (Fig. 5, Supplementary Fig. 8). By contrast, the proportion of N2C type DARPins is remarkably reduced for DANA 3^mod^ and DANA 5^mod^ (Fig. 3e), suggesting that the larger interaction surface of N3C DARPins provided an advantage under these conditions.

Taken together, low stringency selection for affinity in DANA 5^mod^ increased trimer reactivity, but was coupled with a loss of neutralization capacity and, interestingly, a reduction in mutated DARPins. The addition of a V3 pre-panning round prior to ribosome display and the use of a conformationally more stable trimer supported an enrichment of trimer-binding clones which translated into neutralization activity for DANA 1^mod^. The overall neutralization activity was further increased toward trimer, and somewhat counterintuitively, V3-specific neutralization by using trimer preparations with reduced V3-crown exposure in DANA 3^mod^.

### Overcoming the immunogenicity barrier of highly stabilized trimers requires binders with extreme degree of mutations

**Fig. 4:**
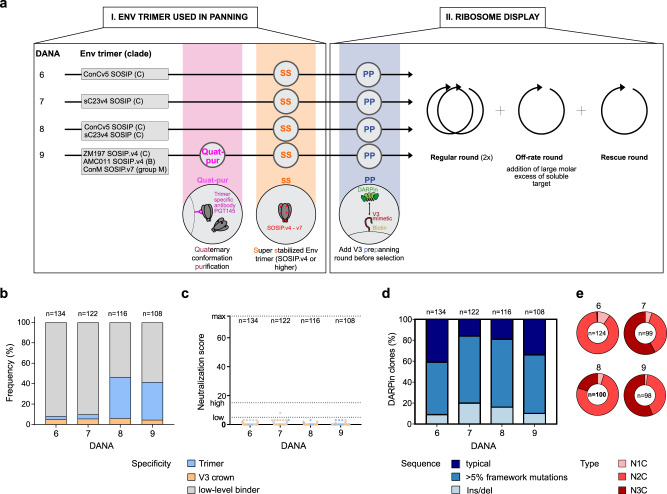
Antigenicity barrier of highly stabilized trimers affords highly mutated binders. **a** DANA 6–9: Overview of recombinant HIV-1 Env trimer used for panning (I) and type and number of ribosome display selection rounds (II). Modifications to ribosome display are indicated. Quat-pur: panning primer purified with PGT145 to ascertain conformation; SS: super-stabilized SOSIP trimer; PP: pre-panning of DARPin library with V3 peptide to remove DARPins highly reactive with V3 before DANA. **b**–**e** DARPin clones with valid ORF derived from randomly picked 190 clones of DANA 6–9 were analyzed for binding, neutralizing and sequence properties (*n* = 108–134). Data are derived from single experiments. **b** Binding properties of DARPins derived in DANA 1–5 based on ELISA using Env trimer probes and the V3-crown mimetic peptide V3-IF (BG505) as specified in Supplementary Table 3. Trimer binding is categorized as binding to at least one of several trimers but not the V3-mimetic. V3 specificity includes solely V3-reactive and V3- and trimer dual-reactive clones. Low-level binders categorize DARPins with no trimer- and V3-reactivity >3-fold over background. **c** Neutralization score against a multi-clade 5-pseudovirus panel reflecting breadth and potency. The max. score is 75, a score 5–14 indicates low neutralizing activity, a score of >15 indicates high neutralizing activity. **d** Frequency of DARPins without mutations (i.e., typical DARPins), with insertions or deletions and with >5% mutations in the framework. **e** Distribution of DARPin types.

Collectively, results from DANA 1–5 screens highlighted a need to reduce immunodominance whilst maintaining affinity maturation. We therefore focused the next series of experiments (DANA 6–9) on assessing several advanced trimer immunogens that were designed for high stability and low V3 exposure in combination with an off-rate selection to enrich high-affinity clones. Two highly stabilized clade C trimers (sC23v4-KIKO and consensus C (ConCv5-KIKO^42^)) were subjected to DANA in individual homotypic screens or in combination (DANA 6–8) (Fig. 4a). In DANA 9, a triple combination of highly stabilized trimers, representing group M (ConM-SOSIP.v7^64^), clade B (AMC011-SOSIP.v4^65^) and clade C (ZM197M-SOSIP.v4^38,66^) was tested.

Results from DANA 6–9 screens confirmed the high degree of stabilization and V3 shielding of these trimers. Hardly any V3 reactive clones were selected (Fig. 4b). Most strikingly, we observed a markedly lower proportion of DARPins with valid ORF compared to DANA 1–5 (Supplementary Fig. 6a). Confirming findings from DANA 1–5, heterotypic screens yielded more trimer-reactive clones than the homotypic regimen. However, for DANA 6–9 the fraction of clones with low-level binding was highest and with the exception of two single DARPin clones in DANA 7, no neutralizing clones were selected (Fig. 4c). These features were paired with a massively increased mutation frequency across all four DANA screens, reaching from 59% mutated in DANA 6 to 84% in DANA 7, with a high proportion of framework mutations compared to DANA 1–5 (Fig. 4d). The distribution of alignment scores and the position-specific mutational analysis suggest that in DANA 6–9 several mutations accumulated in individual DARPins (Supplementary Fig. 6b, c, Supplementary Fig. 7). In line with this finding, a preferential selection of N2C DARPins can be observed for DANA 6–9, predominantly in DANA 6 and DANA 8 performed with ConCv5-KIKO (Fig. 4e). Of note, the DANA 3^mod^ screen was performed in parallel to DANA 6–9 with the identical starting library. Hence, the high accumulation of mutations is intrinsic to DANA 6–9 and not due to differences of the starting library. Taken together, the results from DANA 6–9 confirm that the immunodominant V3 is well shielded on the highly stabilized trimers but also illustrate the difficulty of generating high affinity binders with neutralizing activity from these immunogens. Overall, across all DANA screens we only identified few DARPins that were not V3 directed and harbored at least modest neutralization activity. These were directed against epitopes on open Env as shown by mapping of clones from DANA 5, 3mod and 5mod (Supplementary Fig. 10).

**Fig. 5:**
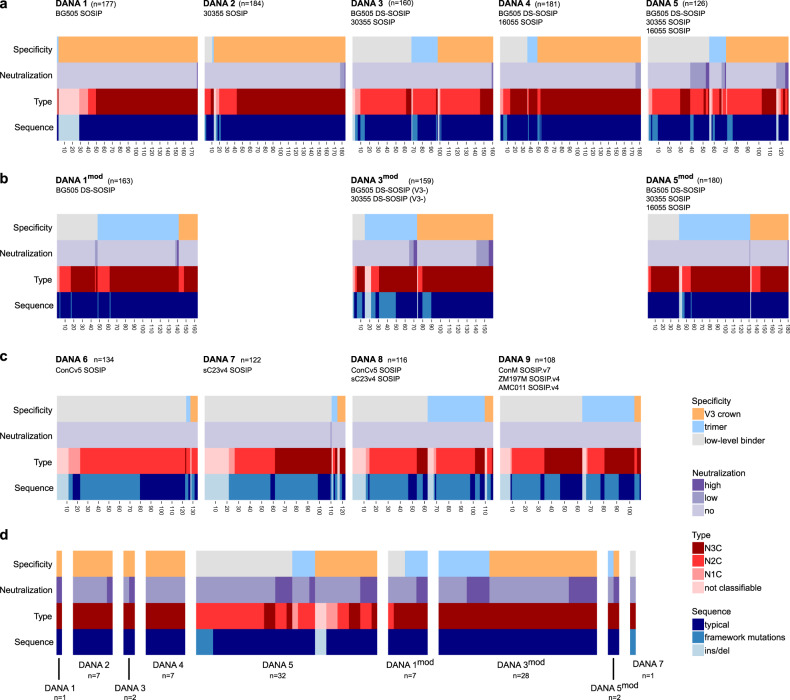
DANA outcome patterns reflect stringency of the Env trimer antigens used as panning target. Complex heatmaps comparing DANA outcome with respect to Env trimer and V3 binding (ELISA), HIV-1 neutralization and DARPin sequence features. **a** DANA 1–5, (**b**), DANA 1mod, 3mod and 5mod and (**c**) DANA 6–9. For each DANA the panning target(s) and the retrieved clones with validated ORF out of 190 randomly picked clones are indicated. **d** Summary of the data from (**a** to **c**) comprising only DARPin clones with neutralizing activity.

A comparison of specificity, neutralizing and sequence parameters of DANA 1–9 individually (Supplementary Fig. 8) and in a complex, categorial heatmap analysis (Fig. 5) illustrates the impact of immunogen shielding on the reactivity pattern in DANA. Subjecting the compiled data from DANA 1–9 to multiple correspondence analysis (MCA) (Supplementary Fig. 11) combined with a stratification into non-mutated and mutated DARPins, we observed three clusters: Cluster 1 is formed by non-mutated DARPins and clusters 2 and 3 comprise mutated DARPins with insertion and deletions or framework mutations, respectively (Supplementary Fig. 11b). DANAs 6–9 showed high mutation frequencies, and the DARPins selected there fell mainly into clusters 2 and 3, while the majority of DARPins from the other DANAs were represented in cluster 1 (Supplementary Fig. 11a). The non-mutated DARPins of cluster 1 harbored the majority of V3 binders, whereas the trimer-binding clones were more evenly distributed in all three clusters, indicating that both trimer-binding clones with and without mutations were selected (Supplementary Fig. 11d). Of particular note, the majority of neutralizing clones fell into clusters 1 and 3, representing both V3 and trimer-binding clones (Supplementary Fig. 11c).

We conclude that DANA captures the limited antigenic properties of closed Env trimers similar to antibodies in natural infection by generating highly mutated binders and thus provides a valuable in vitro tool for the evaluation and selection of immunogens suitable for bnAb induction.

## Discussion

Understanding the counterbalance of epitope shielding and accessibility on Env trimers will be essential to guide immunogen selection for HIV-1 vaccines. To rationalize and dissect this process, we present here a strategy for assessing the antigenicity of immunogens using DARPin technology^44^. In the **D**ARPin **An**tigenicity **A**nalysis (DANA), we explored the capacity of candidate HIV-1 Env trimer immunogens with different degrees of conformational stabilization to select Env-specific and neutralizing DARPin clones. The DANA concept builds on the powerful DARPin selection by ribosome display^47^. Env-specific DARPins are selected from high-content DARPin libraries in consecutive selection rounds against different forms of Env trimers and can be straightforwardly analyzed for features that were selected. By using these artificial binding surfaces orthogonal to antibodies, fundamental principles can be elucidated on how different selection strategies modulate the outcome, independent of particular features of antibodies. We demonstrate that assessing the outcome of a given DANA screen by the proportion of Env binders and neutralizing clones selected and their degree of mutation provides insights into the antigenicity of a given immunogen or immunogen combination (Supplementary Discussion). A key potential of DANA is its ability to detect an immunodominant region within the protein. This can be used to evaluate immunogen combinations in vitro to detect a convergence of responses at the same immunodominant site. DANA offers the possibility to conduct selections on the same or differing immunogens in consecutive rounds, allowing assessment of antigenicity profiles of complex, heterotypic prime-boost regimens in vitro aiding the selection of immunogens.

Extrapolating findings made by DANA to vaccines suggests that a balance between shielding and exposure may be important when designing vaccines and vaccination regimens for bnAb induction, an aspect that is investigated in the frame of guided initiation and maturation of CD4-binding site and fusion-peptide directed Ab vaccine responses^67–70^. Overall, assessment of trimer antigenicity by DANA underscored the need to reduce immunodominant responses to promote evolution of neutralizing responses, recapitulating the experience gained in the development of HIV vaccines in both animal and human trials^1,51,71–73^.

We consider the strength of DANA screens in deriving an antigenicity assessment that can explore fundamental features of binder selection processes during complex immunization protocols. By revealing the impact of distinct antigenic properties of the immunogen on the selection outcome, DANA has the potential to provide information for immunogen selection and design. DANA can, however, not be expected to reveal the exact epitopes recognized by bnAbs, given that the binding properties of DARPins are inherently different from antibodies, due to their design and rigid structure.

Being orthogonal to antibodies, DARPins can decipher very general principles regarding the response to particular antigenic features. As we illustrate here for immunodominant responses and affinity maturation, DANA screens can pinpoint critical steps and install measures that overcome them, but evidently not all measures can be translated to immunization regimens in humans. Adapting the BCR repertoire to reduce precursors reactive with immunodominant clones, in analogy to pre-panning of the DARPin libraries against open Env and V3, cannot be done. There, breaking the immunodominance of counteracting responses in germinal center reactions will be key and research developments leading toward alternative antigen display^74^, antigen delivery including mRNA vaccines^75,76^, and innovative adjuvants^77,78^ will be critical. It will also be crucial to support maturation of initial low-affinity responses directed against neutralization-relevant epitopes. As seen with DANA, initial Env trimer targeting responses induced by vaccination may be of low affinity and may require tailored boosting strategies to increase their affinity while avoiding high-affinity yet non-neutralizing immunodominant responses from taking over^67,79^.

In conclusion, owing to the capacity of DANA screens to recapitulate pitfalls of HIV-1 Env immunogen design, namely high, undesired immunodominance and hyper-shielding, DANA screens can be considered as means for basic antigenicity screening of candidate immunogens with the potential to assist and streamline immunogen selection.

## Methods

### Cells

293 T cells were obtained from the American Type Culture Collection (ATCC) and TZM-bl reporter cells through the NIH AIDS Reagent Program. Both cell lines were cultured in DMEM containing 10% fetal calf serum (FCS) and 100 μg/ml streptomycin and 100 U/ml penicillin (P/S) and were regularly checked for the absence of mycoplasma. HEK 293 T Freestyle^TM^ suspension (293 F) cells (Thermo Fisher) for protein expression were maintained in suspension in serum-free FreeStyle^TM^ 293 F expression media (Thermo Fisher) according to the manufacturer’s instructions.

### V3 peptides

A mixture of V3 mimetics V3-IY (MN) and V3-IF (BG505) was used for pre-panning in specified DANA screens (Supplementary Table 2). Structure-constrained V3-crown mimetic peptides of strains MN and BG505.W6M.ENV.C2 were synthesized as described^80^. The V3 mimetic peptides were designed to build anti-parallel β-strands that differ in the formation of inter-strand hydrogen bonds as described^37^. Peptides were biotinylated for use in ribosome display and ELISA. For V3- IY (MN) and V3-IF (BG505), a PEG08 and a PEG04 linker were introduced between the peptide chain and biotin respectively. All synthetic peptides were ≥95% pure by analytical HPLC and displayed electrospray MS spectra consistent with the expected masses. Sequence information on V3 mimetics and linear V3 peptides can be found in Supplementary Fig. 3.

### HIV envelope trimer proteins

We included a range of Env trimers from different subtypes covering fully closed to partially open trimers (Supplementary Table 1; Supplementary Fig. 2, Supplementary Fig. 3). Trimer preparations were generated by different research groups (Trkola, Sanders/van Gils and Wagner), purified by state-of-the-art protocols by affinity and size-exclusion chromatography to isolate the trimer fraction, and characterized by antigenicity profiling with a panel of Env mAbs (Supplementary Fig. 2, Supplementary Table 5). The BG505-SOSIP source plasmid^3^ was kindly provided by J.P. Moore (Weill Cornell University, New York, USA). 30355 trimers were generated analogous to BG505 SOSIP^3^ and BG505 DS-SOSIP^49^ constructs from an Env cloned from a bnAb inducer identified in the Swiss 4.5 K screen, whose neutralization activity is predicted to comprise several epitope specificities^7,81,82^. BG505 SOSIP, BG505 DS-SOSIP, 30355-SOSIP, and 30355 DS-SOSIP (Supplementary Fig. 2, Supplementary Fig. 3) were produced by co-transfection of HEK 293 T Freestyle suspension (293-F) cells with Env-encoding plasmids and a furin-expressing helper plasmid at a 3:1 ratio as described^83^. Env proteins were purified from culture supernatants using *Galanthus nivalis* lectin resin (Vector Laboratories) as described^84^ and subjected to Superdex 200 size-exclusion chromatography (GE Healthcare, USA) to derive pure monomer or trimer. ConCv5-KIKO and sC23v4-KIKO^42^, ConM-SOSIP.v7^64^, ZM197M-SOSIP.v4^38^, AMC011-SOSIP.v4^38^ and DU422-SOSIP.v4^66^ were expressed similarly and purified as previously described. 16055 SOSIP was produced by R. Wagner with permission from IAVI^85^.

For chemical biotinylation of trimers 16055 SOSIP, sC23v4-KIKO and ConCv5-KIKO NHS-Biotin (Sigma) was dissolved in DMSO at 10 mM concentration. Env trimers were biotinylated at a 1.875 molar excess of NHS-biotin over Env protomers (at >1 mg/ml in PBS). The reaction was allowed to proceed for 45 min at RT, followed by buffer exchange into PBS using Zeba Spin columns (Thermo Fisher Scientific). All other Env proteins were enzymatically biotinylated on a C-terminal AviTag. Proteins were buffer-exchanged into half-concentrated PBS supplemented with 5 mM Mg-acetate, pH 7.5. Proteins were concentrated to 10–40 µM concentration and biotinylated overnight at 37 °C using BirA enzyme (Avidity, Aurora, USA) according to the manufacturer’s instructions.

Env trimers as prepared by the above procedures may contain some partially open trimer proteins. Based on mAb 17b and b12 binding which require considerable opening of the trimer to access their epitopes^50^ trimers 16055 SOSIP and ZM197M SOSIP.v4 were ranked as partially open (Supplementary Fig. 2). Where indicated, the effects of additional purification steps of panning trimers were probed. These were positive selections to enrich for closed trimers in DANA 9 with the V1V2 quaternary structure specific mAb PGT145^38,64^ and negative selections to remove V3-exposing trimers in DANA 3^mod^ with the V3-crown mAb 1–79^86^. For V3 depletion, V3 mAb 1–79 was coupled to protein A agarose resin (Thermo Fisher Scientific) at >3-fold molar excess over BG505-DS-SOSIP or 30355-DS-SOSIP before performing two successive purification rounds with each Env trimer, respectively. Exposure of V3 on the trimer preparations before and after the V3 depletion was monitored by ELISA (Supplementary Fig. 9).

### DARPin selections by Ribosome display

#### Overview

The DARPin technology allows the isolation of target-specific DARPins from original high-content DARPin libraries by ribosome display^47,48^. Ribosome display comprises a transcription, a reverse transcription and a DNA amplification step, which mimics an evolution process, since DNA amplification can be carried out under error-prone conditions. Thus, by generating diversity followed by subsequent biochemical selection for affinity or selectivity, the phenotypes can be shaped toward the desired features.

A single DARPin selection round starts with in vitro transcription and translation of the DARPin library where the nascent DARPin stays linked to the ribosome, as well as its mRNA, and the ribosome is present in a ternary complex (Fig. 1a)^47,48^. In the subsequent panning step, the ternary complexes are incubated with Env-target immobilized on beads. The Env-bound ternary complexes are subsequently disassembled and the mRNA is reverse- transcribed to yield DNA of a DARPin pool enriched in panning-target specific binders, from which the next rounds start (Fig. 1a). Typically, three to four selection rounds are required to yield high-affinity and selective binders^47,48^ (Fig. 1b). A so-called off-rate selection round can improve selection of high-affinity DARPins by adding a large molar excess of non-immobilized antigen to retain clones with low off-rate during selection^47^. An off-rate selection round is commonly followed by a last regular selection round with low-stringency (Fig. 1b) to collect and amplify all high-affinity binders. *E. coli* cells are then transformed with the resulting DARPin pool and screened for the presence of target-specific DARPins with features of interest.

DARPin selections by ribosome display were performed using a 2nd generation N3C DARPin library with and without cap randomization, following the procedures described above^37,47,87,88^ (Fig. 1a, Supplementary Fig. 1). Enzymes used during the ribosome display procedure were T7 RNA polymerase (Thermo Fisher Scientific) for DARPin library transcription, and AffinityScript reverse transcriptase (Agilent) and HerculaseII polymerase (Agilent) for the RT-PCR. To mimic immunization strategies and assess antigenic sites targeted in homotypic and heterotypic prime-boost regimens, different combinations of Env-trimers were used as panning targets (Supplementary Table 2). Supplementary Table 1 provides an overview of the Env-targets employed in the individual selection rounds of each antigenicity screen. The DANA screens were conducted in separate ribosome display runs in the following order: The first run included only DANA 1. In the second run DANA 2, 3, 4, 5, 1^mod^ and 5^mod^ were conducted in parallel, followed by a run including DANA 3^mod^, 6, 7, 8 and 9. DANAs in each run were conducted with the same stock of the 2nd generation N3C library (Supplementary Table 1). Biotinylated Env-derived targets were coupled to magnetic microbeads (MyOne-T1 Dynabeads, Invitrogen) via streptavidin, and panning of ternary DARPin-mRNA-ribosome complexes was performed in 96-well plates on a King Fisher Flex magnetic particle processor (Thermo Fisher Scientific). Ribosome display with decreasing immobilized target concentrations were carried out over five (250 nM, 125 nM, 50 nM, 5 nM, 50 nM) or four rounds (250 nM, 125 nM, 5 nM, 50 nM) for DANA 1 or the other DANAs, respectively. In some antigenicity screens where indicated, in the round with 5 nM target, off-rate selection was performed. For this purpose, after incubation of the ternary complexes with 5 nM target for 1 h, a large molar excess (500 nM) of soluble non-biotinylated target was added and incubated for another hour to select for high-affinity DARPins (off-rate round). In the last round (rescue round), the immobilized target concentration was increased again (50 nM) to amplify the high-affinity DARPins.

To reduce V3 reactivity in the DARPin library, for the indicated screens we conducted a pre-panning round with V3-mimetics (DANA 1^mod^, 3^mod^, and 6–9). The resulting DARPin pool, thus depleted of V3-reactive clones, was then used as starting library for the selections on Env-trimers as described above. DANA 1^mod^ corresponds to DARPin selection V in Glögl et al.^61^ and the first randomly picked 190 clones analyzed there, were re-analyzed in the current study.

### DARPin small-scale purification for binding and neutralization screening

The DARPin pools selected by ribosome display were ligated into a pQE30 (Qiagen) derived expression plasmid in frame with an N-terminal his-tag and a C-terminal FLAG^®^-tag and under the control of a *T5lac* promoter, and *E. coli* XL-1 Blue competent cells were transformed. Next, 190 clones per library were picked for sequencing and further downstream screening for target binding and virus neutralization (Fig. 1a). Only clones with a verified validated ORF (see below) were included in further analysis. For small-scale DARPin protein production, clones were grown in 1 ml in deep-well plates to OD600 = 0.8 before induction of protein expression with 0.5 mM isopropyl-β-D- 1-thiogalactopyranoside (IPTG; Sigma-Aldrich) for 3–4 h. Bacterial pellets were lysed in 50 µl B-PER II (Thermo Fisher Scientific), shaken for 15 min at 1300 rpm on an orbital microtiterplate shaker, and then incubated without shaking for 50 min at RT. 1 ml TBS/0.1% Tween/500 mM NaCl/0.1% BSA (pH 8) was added and the equilibrated lysate was centrifuged (1500 *g*, 20 min, 4 °C) to remove cell debris. The supernatant was transferred to HisPur™ Cobalt Spin Plates (Thermo Fisher Scientific) and purified according to the manufacturer’s instructions. Afterwards, the DARPin eluate was transferred to AcroPrep 96 filter plates (Pall, 3 kDa cut-off) for an additional buffer-exchange, in which imidazole was removed by washing three times with 150 µl PBS. From these small-scale purifications, DARPin eluates were used directly without concentration adjustment for the initial binding and neutralization screens.

### Detection of DARPin and mAb binding by ELISA

High-binding microplates (Corning) were coated with 60 nM Neutravidin (Thermo Fisher Scientific), either overnight at 4 °C or for 1 h at room temperature (RT). Following that, all steps were performed at RT. Free binding sites were blocked with TBS-TB (Tris-buffered saline containing 0.1% Tween-20 (pH 7.5, Sigma Aldrich) and 1% bovine serum albumin (Sigma Aldrich)) for 1 h, before immobilizing the biotinylated target proteins or peptides at a final concentration of 20 nM for 1 h. After three washing steps with TBS-T (Tris-buffered saline containing 0.1% Tween-20), serial dilutions of purified DARPins or mAbs (Supplementary Table 5) were added in TBS-TB. Unbound material was washed off with TBS-T and DARPins were detected via their FLAG^®^ tag using a mouse monoclonal anti-FLAG^®^ antibody (Sigma Aldrich, clone M2, Cat#F1804 or Cat#F3165) and a goat anti-mouse IgG Alkaline Phosphatase secondary antibody (Sigma Aldrich, Cat#A3562) at a final dilution of 1:15’000 each. Binding of mAbs was detected using polyclonal goat anti-human IgG (Fc specific) alkaline phosphatase-conjugated antibody (Sigma-Aldrich, Cat#A9544) diluted 1:60’000 in TBS-TB. Emission of relative light units was measured after addition of Tropix CDP-star chemiluminescent substrate (Tropix CDP-star, Thermo Fisher Scientific) on a Dynex Technologies Luminometer. A list of protein targets screened by ELISA is shown in Supplementary Table 3. DARPins with a valid ORF (i.e., with the start and stop sequence in frame (Supplementary Fig. 1) and no stop codon within the ORF) were screened for their binding specificity (see below). DARPins with no trimer and V3 binding >3-fold above neutravidin background binding were ranked as low-level binders.

Competition binding experiments were performed using a similar setup with V1V2-deleted trimeric BG505-SOSIP trimer immobilized to neutravidin via a biotinylated C-terminal avi-tag as target. Competing agents were preincubated with the target for 1 h at saturating concentration (bnD.2: 0.5 µM, bnD.8: 10 nM, VRC01: 2 µg/mL, F425-B4e8: 0.5 µg/mL, 17b: 2 µg/mL). DARPins were added at saturating concentration (103.2 G07: 0.5 µM; 105.1 A01, 105.1 C01,and 105.1 C12: 1 µM; 105.1 G11: 10 µM; 209.1 G08, 209.2 B11, 209.2 D02, 209.2 E03, and 209.2 E08: 10 nM) and allowed to bind for an additional hour before washing three times with TBS-T. DARPin binding in presence or absence of competitor was detected with mouse anti-FLAG mAb and competition was expressed relative to the signal obtained without competitor.

### Neutralization assay

Inhibitory activity against pseudoviruses was determined using the Env pseudovirus luciferase reporter assay on TZM-bl^89^. For the production of Env pseudovirus stocks, 293 T cells were transfected with the luciferase reporter HIV-1 pseudotype vector pNLlucAM and env expression plasmids^89^. Env genes were expressed from pcDNA3.1 expression vectors. A full list of Env pseudotyped viruses, generated with corresponding GenBank entry and subtype, is provided in Supplementary Table 4. In the neutralization screen, inhibitory capacity of 190 DARPin clones per DANA was assessed in a 384-well neutralization assay format as described previously^89^. Briefly, purified DARPins from small-scale purification diluted at 1:6 with DMEM were preincubated with the respective virus for 1 h, before infection of target cells. Virus input was chosen to yield virus infectivity corresponding to 5000–20,000 relative light units (RLU) in the absence of inhibitors as measured on a Dynex MLX instrument. Details on the virus panel are listed in Supplementary Table 3 and Supplementary Table 4. The previously identified broadly neutralizing DARPin bnD.3 was prepared in parallel and included as positive control^37^. Luminescence readouts were obtained on a Perkin Elmer EnVision Multilabel Reader. To evaluate breadth and potency, we used a scoring algorithm of the neutralization activity measured on the 5-pseudovirus panel. Neutralization activity of each DARPin against a specific virus received a score of 0 when neutralization activity was below 50%, a score of 1 when neutralization activity ranged between 50% and <70%, a score of 2 for neutralization between 70% and <90% and a score of 3 for neutralization ≥90%. Neutralizing activity was further multiplied with neutralization breadth (1 point per virus neutralized with an activity of above 50%), which yields a maximum cumulative neutralization score of 75. Clones with a score of 4–15 were classified as weakly neutralizing, clones with a score of >15 were considered highly neutralizing (Supplementary Fig. 4). DARPins with a valid ORF were screened for their neutralization ability (see below).

### DARPin sequence analysis

190 DARPin clones per DANA screen were sequenced using DNA Sanger sequencing (Microsynth AG, Balgach, Switzerland). The sequences were analyzed using *emboss* (v6.6.0)^90^, *bioawk* (v1.0), *seqtk* (v1.3), *seqkit* (v0.10.1)^91^ and the results summarized and visualized using R (v4.2.1). First, raw sequencing data (AB1) were converted to FASTQ using *seqret*, and low-quality bases were trimmed from both ends using *seqtk trimfq* with an error rate threshold of 0.05. Then the DARPin ORF was verified. A valid DARPin ORF was defined as a DARPin sequence containing a 15 nucleotide-long sequence stretch containing the start codon (**ATG**AGAGGATCGCAT), and a 15 nucleotide-long sequence stretch containing the stop codon (GACGACGACAAG**TAA**) (Supplementary Fig. 1), as both the start and stop sequence stretches are part of the DARPin expression vector. These sequence stretches are fully conserved, as they are not affected by random mutagenesis during ribosome display selection, because they are upstream of the BamHI and downstream of the HindIII DARPin-pool cloning sites, respectively. Only DARPin sequences/clones with a valid ORF (intact start or stop sequences and no pre-mature stop) were included in further analysis. DARPin clones with a valid ORF were then investigated for their amino acid sequence length. DARPins may occasionally reduce their length by dropping an internal repeat through recombination during the PCR inherent in the ribosome display procedure^60^. The expected length for DARPins is 117, 150 or 183 residues for one (N1C), two (N2C) or three (N3C) internal repeats, respectively. DARPins that could not be classified in one of these types (+/-1 amino acid), were considered to have mutations in the form of insertions or deletions. DARPin clones with a valid ORF and a N1C, N2C or N3C type were further analyzed for mutations, in the non-randomized residues constituting the DARPin protein framework, responsible for their structure and stability. A pairwise alignment of each DARPin to a respective consensus sequence for each DARPin type was performed using *needleall*^90^. To retrieve a measure of mutations that is similar to GLI for antibodies, we calculated an alignment score (Fig. 2c, Supplementary Fig. 6c). The higher the alignment score, the closer the sequence is to the consensus sequence. To determine the percentage of mutated framework residues for individual DARPins, the highest alignment score obtained among all DARPins aligned to the consensus sequence was set as 100% and from this the individual percentage of alignment was calculated. We nominally set alignment of 95% of the framework residues of an individual DARPin sequence to the reference sequence as threshold for an intact framework. To allow a more detailed DARPin-specific mutation analysis, we further recorded the number and position of amino acid substitutions, compared to the same consensus sequences used for the alignment score as shown in Supplementary Fig. 7.

### Human specimens and ethics

In the current study, biobanked peripheral blood mononuclear cells (PBMC) from 21 samples of 19 HIV-1 infected individuals, identified as bnAb inducers in the *Swiss 4.5* *K Screen*^7,11^, were utilized to study Env-specific B cell receptors (BCRs). PBMC samples were derived from specimens stored in the biobank of the Swiss HIV Cohort study (SHCS)^82^ which covers their use in the current study. The SHCS is registered under the Swiss National Science longitudinal platform: https://www.snf.ch/en/YA2SxeDV03G25gyJ/funding/programmes/longitudinal-studies. The SHCS and the use of its biobanked material has been approved by the ethics committee of the participating institutions (Kantonale Ethikkommission Bern, Ethikkommission des Kantons St. Gallen, Comité départemental d’éthique des spécialités médicales et de médicine communautaire et de premier recours, Kantonale Ethikkommission Zürich, Repubblica e Cantone Ticino - Comitato Ethico Cantonale, Commission cantonale d’éthique de la recherche sur l'être humain, Ethikkommission beider Basel for the SHCS and Kantonale Ethikkommission Zürich for the ZPHI) and written informed consent had been obtained from all participants.

### Preparation of memory B cells for single cell BCR sequencing

Cryo-preserved peripheral blood mononuclear cells (PBMC, ~10^7^ cells) from previously identified HIV-1 bnAb inducers were obtained from the SHCS biobank^7^. Cells were thawed, washed, and stained in PBS with 2% FCS with fluorescently labelled antibodies for enriching B cells by sorting and TotalseqC barcode-labelled probes and antibodies for LIBRA-seq (Linking B cell receptor to an antigen)^56^ profiling. Fluorescently labelled mouse monoclonal antibodies (APC-Cy7 conjugated antibodies against human CD16 (clone 3G8), CD14 (clone HCD14), CD8 (clone SK1), and CD3 (clone SK7) as well as APC conjugated antibody against human IgD (clone IA6–2) and Brilliant Violet 421 conjugated antibody against human CD19 (clone HIB19)) and TotalseqC barcode-labelled antibodies to CD21 (C0181, clone Bu32), CD27 (C0154, clone O323) and CD38 (C0410, clone HB-7) were purchased from Biolegend, San Diego CA. For TotalseqC barcoding of HIV-1 antigens, biotinylated HIV-1 antigens were incubated with TotalseqC streptavidin-PE (Biolegend, San Diego CA) overnight at room temperature according to the manufacturer’s instructions and then stored at 4 °C for up to 30 days. TotalseqC barcode labelled antigens included soluble, stabilized HIV-1 Env trimers (AMC011 SOSIP.v4, ConM SOSIP.v7, DU422 SOSIP.v4) and linear V3 peptides (V3_JRFL, V3_BG505) (see Supplementary Fig. 3 for V3 sequences). Fluorescently labelled mAbs and TotalseqC labelled PBMC were incubated for 30 min at room temperature, washed (PBS with 2% FCS) and CD19 positive, IgD negative B cells (comprising memory B cells and plasmablasts) were sorted on a BD FACSAria™ III System (Beckton Dickinson, USA) (see Supplementary Fig. 5a).

### 10X genomics BCR and LIBRA-seq library preparation

Freshly sorted B cells were prepared for loading on the Chromium Controller microfluidics device (10X Genomics, USA) according to the user manual CG000208 “Chromium NextGEM SingleCell V(D)J Reagent Kits v1.1 with Feature Barcode technology for Cell Surface Protein”. A maximum of 15,000 cells were loaded per lane on a Next GEM Chip G (10X Genomics). Sample preparation for the cellular mRNA library and LIBRA-seq barcode library, containing the B cell receptor (BCR) and antigen binding information respectively, was performed according to the user manual. Obtained BCR and LIBRA-seq barcode libraries (CD38, CD27, CD21, HIV-1 Env antigens) were sequenced on a NovaSeq 6000 (Illumina, San Diego, USA).

Raw output files were processed using 10X Genomics CellRanger 4.0.0. BCR, and TotalseqC barcode libraries were processed separately. Downstream data analysis for the BCR library was performed using the Immcantation Framework (http://immcantation.org) with Change-O v1.2.0^92^. Briefly, IgBlast v. 1.19.0 was used to annotate the all_contig.fasta file against the IMGT database, combined with information from the all_contig_annotations.csv file and split into separate files for BCR heavy and light chains. Only productive sequences with unique molecular identifier (UMI) counts ≥5 were included for further analysis. Heavy chains were assigned into clonal groups using hierarchical clustering. The clustering threshold of the junction distance was determined with the SHazaM R package for each sample^92^. Heavy chain clonotypes were refined based on light chain information and cell duplets were corrected by only keeping the sequence with the highest UMI count using the Change-O light_cluster.py script. Feature barcode libraries were filtered for cells with total counts >50. Separate files of heavy chains, light chains and TotalseqC barcode information were combined based on the cell barcode using R v4.2.1.

In total 21 PBMC samples from 19 HIV-1 bnAb inducers were included in the analysis shown in Fig. 2a and Supplementary Fig. 5^7^. Across all samples this yielded a total of 80,963 IgG and IgA BCRs. BCRs were categorized depending on their capacity to bind to TotalseqC labelled HIV-1 Env trimers and linear V3 constructs. Trimer-binding BCR: binds at least one of three trimers (AMC011_SOSIP.v4, ConM_SOSIP.v7, DU422_SOSIP.v4). V3-binding BCR: binds at least one of two V3 peptides (V3_BG505 or V3_JRFL). Thresholds for each protein or peptide were set at the 99.5th percentile. BCRs above this cut-off were categorized as trimer- or V3-reactive, respectively.

### Antigenic characterization of Env trimers

Antigenic characterization was performed as described previously using a bead-based multiplexed immunoassay using the Luminex technology^©^^93^ (Supplementary Fig. 2). In brief, carboxylated MagPlex© beads (Luminex) were coupled with Neutravidin (Sigma Aldrich) followed by loading with biotinylated antigens. Coupling was done using a coupling kit (BioRad) according to the manufacturer’s instructions and used as described. The bead regions with the different antigens were mixed and incubated with monoclonal Abs (Supplementary Table 5) diluted in PBS-BSA 1% overnight at 4 °C. IgG responses were detected with phycoerythrin (PE)-labeled secondary mouse antibody specific for human IgG-Fc (Southern Biotech, Cat#9040–09, clone JDC-10) diluted in PBS-BSA 1% at a concentration of 1 μg/ml for 1 h at room temperature. After extensive washing with PBS/BSA 1% beads were analyzed with the FlexMap 3D or LX200 instruments (Luminex). A minimum of 50 beads per antigen and plasma were acquired to guarantee accurate MFI values. For 16055 SOSIP, antigenic characterization was performed by ELISA as described above, due to difficulties in detection with flow-based Luminex technology^©^.

### Statistical and graphical analyses

Statistical analyses were performed in R (v4.2.0) and using Prism 7 (GraphPad Software). Complex heatmaps (Fig. 5) were established in R^94^.

### Supplementary information

Maliqi et al_Supplementary Information

Reporting-summary

Supplementary dataset for Figure 1

Supplementary dataset for Figure 2 and Supplementary Figure 5c

Supplementary dataset for Figure 3

Supplementary dataset for Figure 4

Supplementary dataset for Figure 5 and Supplementary Figures 8 and 11

Supplementary dataset for Supplementary Figure 4

Supplementary dataset for Supplementary Figure 5b

Supplementary dataset for Supplementary Figure 6

Supplementary dataset for Supplementary Figure 7

Supplementary dataset for Supplementary Figure 9

## Data Availability

Source data for all displayed items are provided as Supplementary Datasets. Raw datasets underlying the source data are available from the corresponding author on request.

## References

[1] Ng’uni T, Chasara C, Ndhlovu ZM (2020). Major scientific hurdles in HIV vaccine development: Historical perspective and future directions. Front. Immunol..

[2] Hargrave, A., Mustafa, A. S., Hanif, A., Tunio, J. H. & Hanif, S. N. M. Current status of HIV-1 vaccines. *Vaccines (Basel)* **9**, 10.3390/vaccines9091026 (2021). 10.3390/vaccines9091026 PMC8471857 34579263

[3] Sanders RW (2013). A next-generation cleaved, soluble HIV-1 Env trimer, BG505 SOSIP.664 gp140, expresses multiple epitopes for broadly neutralizing but not non-neutralizing antibodies. PLoS Pathog..

[4] Yasmeen A (2014). Differential binding of neutralizing and non-neutralizing antibodies to native-like soluble HIV-1 Env trimers, uncleaved Env proteins, and monomeric subunits. Retrovirology.

[5] Guttman M (2015). Antibody potency relates to the ability to recognize the closed, pre-fusion form of HIV Env. Nat. Commun..

[6] Subbaraman H, Schanz M, Trkola A (2018). Broadly neutralizing antibodies: What is needed to move from a rare event in HIV-1 infection to vaccine efficacy?. Retrovirology.

[7] Rusert P (2016). Determinants of HIV-1 broadly neutralizing antibody induction. Nat. Med..

[8] Abela IA, Kadelka C, Trkola A (2019). Correlates of broadly neutralizing antibody development. Curr. Opin. HIV AIDS.

[9] Kouyos RD (2018). Tracing HIV-1 strains that imprint broadly neutralizing antibody responses. Nature.

[10] Landais E, Moore PL (2018). Development of broadly neutralizing antibodies in HIV-1 infected elite neutralizers. Retrovirology.

[11] Kadelka C (2018). Distinct, IgG1-driven antibody response landscapes demarcate individuals with broadly HIV-1 neutralizing activity. J. Exp. Med..

[12] Rantalainen K (2018). Co-evolution of HIV Envelope and Apex-Targeting Neutralizing Antibody Lineage Provides Benchmarks for Vaccine Design. Cell Rep..

[13] Liao HX (2013). Co-evolution of a broadly neutralizing HIV-1 antibody and founder virus. Nature.

[14] Bonsignori M (2017). Antibody-virus co-evolution in HIV infection: paths for HIV vaccine development. Immunol. Rev..

[15] Havenar-Daughton C, Lee JH, Crotty S (2017). Tfh cells and HIV bnAbs, an immunodominance model of the HIV neutralizing antibody generation problem. Immunol. Rev..

[16] Kepler TB (2014). Immunoglobulin gene insertions and deletions in the affinity maturation of HIV-1 broadly reactive neutralizing antibodies. Cell Host Microbe.

[17] Klein F (2013). Somatic mutations of the immunoglobulin framework are generally required for broad and potent HIV-1 neutralization. Cell.

[18] Wiehe K (2018). Functional relevance of improbable antibody mutations for HIV broadly neutralizing antibody development. Cell Host Microbe.

[19] Sok D (2013). The effects of somatic hypermutation on neutralization and binding in the PGT121 family of broadly neutralizing HIV antibodies. PLoS Pathog..

[20] Liu M (2015). Polyreactivity and autoreactivity among HIV-1 antibodies. J. Virol..

[21] Prigent J (2018). Conformational plasticity in broadly neutralizing HIV-1 antibodies triggers polyreactivity. Cell Rep..

[22] Wu X, Kong XP (2016). Antigenic landscape of the HIV-1 envelope and new immunological concepts defined by HIV-1 broadly neutralizing antibodies. Curr. Opin. Immunol..

[23] Victora GD, Nussenzweig MC (2022). Germinal centers. Annu. Rev. Immunol..

[24] Roskin KM (2020). Aberrant B cell repertoire selection associated with HIV neutralizing antibody breadth. Nat. Immunol..

[25] Moore PL (2006). Nature of nonfunctional envelope proteins on the surface of human immunodeficiency virus type 1. J. Virol..

[26] Griffith SA, McCoy LE (2021). To bnAb or not to bnAb: Defining broadly neutralising antibodies against HIV-1. Front. Immunol..

[27] Shulman Z (2014). Dynamic signaling by T follicular helper cells during germinal center B cell selection. Science.

[28] Havenar-Daughton C (2016). Direct probing of germinal center responses reveals immunological features and bottlenecks for neutralizing antibody responses to HIV env trimer. Cell Rep..

[29] Dal Porto JM, Haberman AM, Kelsoe G, Shlomchik MJ (2002). Very low affinity B cells form germinal centers, become memory B cells, and participate in secondary immune responses when higher affinity competition is reduced. J. Exp. Med..

[30] Schwickert TA (2011). A dynamic T cell-limited checkpoint regulates affinity-dependent B cell entry into the germinal center. J. Exp. Med..

[31] Shih TAY, Meffre E, Roederer M, Nussenzweig MC (2002). Role of BCR affinity in T cell-dependent antibody responses in vivo. Nat. Immunol..

[32] Burton DR, Mascola JR (2015). Antibody responses to envelope glycoproteins in HIV-1 infection. Nat. Immunol..

[33] Tomaras GD, Haynes BF (2009). HIV-1-specific antibody responses during acute and chronic HIV-1 infection. Curr. Opin. HIV AIDS.

[34] Chen B (2019). Molecular mechanism of HIV-1 entry. Trends Microbiol..

[35] Rusert P (2011). Interaction of the gp120 V1V2 loop with a neighboring gp120 unit shields the HIV envelope trimer against cross-neutralizing antibodies. J. Exp. Med..

[36] Moore PL, Gray ES, Morris L (2009). Specificity of the autologous neutralizing antibody response. Curr. Opin. Hiv. Aids.

[37] Friedrich N (2021). Distinct conformations of the HIV-1 V3 loop crown are targetable for broad neutralization. Nat. Commun..

[38] de Taeye SW (2015). Immunogenicity of stabilized HIV-1 envelope trimers with reduced exposure of non-neutralizing epitopes. Cell.

[39] de Taeye SW (2018). Stabilization of the gp120 V3 loop through hydrophobic interactions reduces the immunodominant V3-directed non-neutralizing response to HIV-1 envelope trimers. J. Biol. Chem..

[40] Torrents de la Pena A, Sanders RW (2018). Stabilizing HIV-1 envelope glycoprotein trimers to induce neutralizing antibodies. Retrovirology.

[41] Kulp DW (2017). Structure-based design of native-like HIV-1 envelope trimers to silence non-neutralizing epitopes and eliminate CD4 binding. Nat. Commun..

[42] Hauser, A. et al. Stepwise conformational stabilization of a HIV-1 clade C consensus envelope trimer immunogen impacts the profile of vaccine-induced antibody responses. *Vaccines (Basel)* **9**, 10.3390/vaccines9070750 (2021). 10.3390/vaccines9070750 PMC8310183 34358165

[43] Jardine J (2013). Rational HIV immunogen design to target specific germline B cell receptors. Science.

[44] Plückthun A (2015). Designed ankyrin repeat proteins (DARPins): binding proteins for research, diagnostics, and therapy. Annu. Rev. Pharm. Toxicol..

[45] Binz HK (2004). High-affinity binders selected from designed ankyrin repeat protein libraries. Nat. Biotechnol..

[46] Binz HK, Stumpp MT, Forrer P, Amstutz P, Plückthun A (2003). Designing repeat proteins: well-expressed, soluble and stable proteins from combinatorial libraries of consensus ankyrin repeat proteins. J. Mol. Biol..

[47] Dreier B, Plückthun A (2012). Rapid selection of high-affinity binders using ribosome display. Methods Mol. Biol..

[48] Plückthun A (2012). Ribosome display: a perspective. Methods Mol. Biol..

[49] Kwon YD (2015). Crystal structure, conformational fixation and entry-related interactions of mature ligand-free HIV-1 Env. Nat. Struct. Mol. Biol..

[50] Ozorowski G (2017). Open and closed structures reveal allostery and pliability in the HIV-1 envelope spike. Nature.

[51] Sanders RW (2015). HIV-1 VACCINES. HIV-1 neutralizing antibodies induced by native-like envelope trimers. Science.

[52] Parker Miller, E., Finkelstein, M. T., Erdman, M. C., Seth, P. C. & Fera, D. A structural update of neutralizing epitopes on the HIV envelope, a moving target. *Viruses* **13**, 10.3390/v13091774 (2021). 10.3390/v13091774 PMC8472920 34578355

[53] Burton DR, Hangartner L (2016). Broadly neutralizing antibodies to HIV and their role in vaccine design. Annu. Rev. Immunol..

[54] Bonsignori M (2016). Maturation pathway from germline to broad HIV-1 neutralizer of a CD4-mimic antibody. Cell.

[55] Bonsignori, M. et al. Staged induction of HIV-1 glycan-dependent broadly neutralizing antibodies. *Sci. Transl. Med.* **9**, 10.1126/scitranslmed.aai7514 (2017). 10.1126/scitranslmed.aai7514 PMC5562350 28298420

[56] Setliff I (2019). High-throughput mapping of B cell receptor sequences to antigen specificity. Cell.

[57] Pejchal R (2011). A potent and broad neutralizing antibody recognizes and penetrates the HIV glycan shield. Science.

[58] Stanfield RL (2020). Structural basis of broad HIV neutralization by a vaccine-induced cow antibody. Sci. Adv..

[59] Schilling J, Schöppe J, Plückthun A (2014). From DARPins to LoopDARPins: novel LoopDARPin design allows the selection of low picomolar binders in a single round of ribosome display. J. Mol. Biol..

[60] Amstutz P, Koch H, Binz HK, Deuber SA, Plückthun A (2006). Rapid selection of specific MAP kinase-binders from designed ankyrin repeat protein libraries. Protein Eng. Des. Sel..

[61] Glögl M (2023). Trapping the HIV-1 V3 loop in a helical conformation enables broad neutralization. Nat. Struct. Mol. Biol..

[62] Ringe, R. P. et al. Reducing V3 antigenicity and immunogenicity on soluble, native-like HIV-1 env SOSIP trimers. *J. Virol*. **91**, 10.1128/JVI.00677-17 (2017). 10.1128/JVI.00677-17 PMC5512241 28539451

[63] Ward AB, Wilson IA (2017). The HIV-1 envelope glycoprotein structure: nailing down a moving target. Immunol. Rev..

[64] Sliepen K (2019). Structure and immunogenicity of a stabilized HIV-1 envelope trimer based on a group-M consensus sequence. Nat. Commun..

[65] van Gils MJ (2016). An HIV-1 antibody from an elite neutralizer implicates the fusion peptide as a site of vulnerability. Nat. Microbiol..

[66] Julien JP (2015). Design and structure of two HIV-1 clade C SOSIP.664 trimers that increase the arsenal of native-like Env immunogens. Proc. Natl Acad. Sci. USA.

[67] Dubrovskaya V (2019). Vaccination with glycan-modified HIV NFL envelope trimer-liposomes elicits broadly neutralizing antibodies to multiple sites of vulnerability. Immunity.

[68] Jardine JG (2016). Minimally mutated HIV-1 broadly neutralizing antibodies to guide reductionist vaccine design. PLoS Pathog..

[69] Xu K (2018). Epitope-based vaccine design yields fusion peptide-directed antibodies that neutralize diverse strains of HIV-1. Nat. Med..

[70] Kong R (2019). Antibody lineages with vaccine-induced antigen-binding hotspots develop broad HIV neutralization. Cell.

[71] Montefiori DC (2012). Magnitude and breadth of the neutralizing antibody response in the RV144 and Vax003 HIV-1 vaccine efficacy trials. J. Infect. Dis..

[72] Bianchi M (2018). Electron-Microscopy-based epitope mapping defines specificities of polyclonal antibodies elicited during HIV-1 BG505 envelope trimer immunization. Immunity.

[73] Sok D (2017). Rapid elicitation of broadly neutralizing antibodies to HIV by immunization in cows. Nature.

[74] Karch CP, Matyas GR (2021). The current and future role of nanovaccines in HIV-1 vaccine development. Expert Rev. Vacc..

[75] Zhang P (2021). A multiclade env-gag VLP mRNA vaccine elicits tier-2 HIV-1-neutralizing antibodies and reduces the risk of heterologous SHIV infection in macaques. Nat. Med..

[76] Mu, Z., Haynes, B. F. & Cain, D. W. HIV mRNA vaccines-progress and future paths. *Vaccines (Basel)* **9**, 10.3390/vaccines9020134 (2021). 10.3390/vaccines9020134 PMC7915550 33562203

[77] Sliepen K (2021). Interplay of diverse adjuvants and nanoparticle presentation of native-like HIV-1 envelope trimers. NPJ Vacc..

[78] Rao M, Alving CR (2016). Adjuvants for HIV vaccines. Curr. Opin. HIV AIDS.

[79] Jardine JG (2015). HIV-1 VACCINES. Priming a broadly neutralizing antibody response to HIV-1 using a germline-targeting immunogen. Science.

[80] Riedel T (2011). Synthetic virus-like particles and conformationally constrained peptidomimetics in vaccine design. Chembiochem.

[81] Scherrer AU (2022). Cohort profile update: The Swiss HIV Cohort Study (SHCS). Int J. Epidemiol..

[82] Schoeni-Affolter F (2010). Cohort profile: the Swiss HIV Cohort study. Int J. Epidemiol..

[83] Binley JM (2002). Enhancing the proteolytic maturation of human immunodeficiency virus type 1 envelope glycoproteins. J. Virol..

[84] Selvarajah S (2005). Comparing antigenicity and immunogenicity of engineered gp120. J. Virol..

[85] Guenaga J (2015). Well-ordered trimeric HIV-1 subtype B and C soluble spike mimetics generated by negative selection display native-like properties. PLoS Pathog..

[86] Scheid JF (2009). Broad diversity of neutralizing antibodies isolated from memory B cells in HIV-infected individuals. Nature.

[87] Mann A (2013). Conformation-dependent recognition of HIV gp120 by designed ankyrin repeat proteins provides access to novel HIV entry inhibitors. J. Virol..

[88] Zahnd C, Amstutz P, Plückthun A (2007). Ribosome display: selecting and evolving proteins in vitro that specifically bind to a target. Nat. Methods.

[89] Mann AM (2009). HIV sensitivity to neutralization is determined by target and virus producer cell properties. Aids.

[90] Rice P, Longden I, Bleasby A (2000). EMBOSS: the European Molecular Biology Open Software Suite. Trends Genet..

[91] Shen W, Le S, Li Y, Hu F (2016). SeqKit: A cross-platform and ultrafast toolkit for FASTA/Q file manipulation. PLoS ONE.

[92] Gupta NT (2015). Change-O: a toolkit for analyzing large-scale B cell immunoglobulin repertoire sequencing data. Bioinformatics.

[93] Liechti T (2018). Development of a high-throughput bead based assay system to measure HIV-1 specific immune signatures in clinical samples. J. Immunol. Methods.

[94] Gu, Z. Complex heatmaps reveal patterns and correlations multidimensional Genomic data. 10.1093/bioinformatics/btw313. (2016). 10.1093/bioinformatics/btw313 27207943

