# Supplementary material for: Assessing immunogenicity barriers of the HIV-1 envelope trimer
Source: NPJ Vaccines. 2023 Sep 30;8:148. doi: 10.1038/s41541-023-00746-3 (PMC10542815; doi:10.1038/s41541-023-00746-3)
Supplement: Supplementary file 1 — Maliqi et al_Supplementary Information [file 41541_2023_746_MOESM1_ESM.pdf]

Supplementary Information

This Supplementary Information is submitted by the authors to provide additional information about their work.

Supplement to: Liridona Maliqi[1]#, Nikolas Friedrich[1]#, et al., “Assessing immunogenicity barriers of the HIV-1 envelope trimer”

# contributed equally

Supplementary Information to Manuscript Entitled

"Assessing immunogenicity barriers of the HIV-1 envelope trimer"

Table of Contents

Supplementary Discussion 3
References to Supplementary Discussion 5
Supplementary Figures 7
Supplementary Figure 1: Schematic of the Open Reading Frame (ORF) of an N3C DARPin 7
Supplementary Figure 2: Antigenic characterization of panning trimers 8
Supplementary Figure 3: Alignment of V3 loop sequences 9
Supplementary Figure 4: Neutralization screening and scoring for DANA 1 10
Supplementary Figure 5: Target-specific analysis of variable heavy (VH) chain germline identities in bnAb inducers 11
Supplementary Figure 6: Sequencing analysis 13
Supplementary Figure 7: Analysis of position-specific substitutions within the DARPin framework 15
Supplementary Figure 8: Binding properties and neutralization capacities associated with DARPin type and sequence integrity 16
Supplementary Figure 9: Depletion of trimers with exposed V3-loop 17
Supplementary Figure 10: Epitope mapping of several DARPins with virus neutralizing activity 18
Supplementary Figure 11: Multiple Correspondence Analysis 19
Supplementary Tables 21
Supplementary Table 1: Env targets used for ribosome display 21
Supplementary Table 2: Overview of panning targets used in ribosome display rounds in DANA 1-9 22
Supplementary Table 3: Neutralization and binding screen 23
Supplementary Table 4: Env-pseudovirus panel 24
Supplementary Table 5: Antibody source 25

Supplementary Discussion

Despite advances in HIV prevention and treatment, an HIV vaccine that provides protective immunity through induction of bnAbs is urgently needed to halt the pandemic[1,2]. Soluble, recombinant, native-like Env trimers stabilized in the closed prefusion trimer conformation presenting bnAb epitopes and preventing exposure of immunodominant non-neutralizing epitopes are considered a critical component of bnAb inducing vaccines both as prime or boost immunogens[3,4]. While ongoing efforts to stabilize HIV-1 Env trimer immunogens have greatly reduced the induction of undesirable antibody responses, such as non-neutralizing V3-crown antibodies, they have failed to induce the desired potent, and broad HIV-1 neutralization of tier-2 viruses representative for the majority of circulating strains[5,6]. Understanding the counterbalance of epitope shielding and required accessibility on Env trimers will therefore be essential to guide immunogen selection for HIV-1 vaccines.

Here, we subjected Env trimers with varying degree of stabilization and V3 exposure to DANA. Some residual V3 exposure is common and observed even on highly stabilized Env trimers[7-9]. Notably, despite extensive stabilization and purification to ascertain the structural homogeneity of trimer immunogens, trimers may disassemble in vivo, exposing unwanted regions such as the V3[10,11]. The V3-crown is a dominant epitope targeted by the DARPin system as also noted in earlier studies[12-14]. While the DARPin library is very diverse and can accommodate a wide range of targets[15], DARPins are not known to bind glycans, and the rigid concave target binding surface of DARPins may lead to preferences for certain epitopes due to shape complementarity. The dominance of V3-crown-reactive DARPins is likely a consequence of binding properties of DARPins and the Env trimer as complex target that efficiently shields most epitopes through glycosylation and conformational masking, rendering the partially exposed V3 as main targetable site. Since avoiding non-neutralizing V3 responses in vaccines is critical, we used DANA screens to specifically investigate ways to break the V3 dominance and the overall consequence of these actions.

In DANA 1, the comparatively less stabilized BG505-SOSIP trimer predominantly led to the selection of V3-reactive DARPins paired with low trimer reactivity, which also has been frequently observed in HIV Env vaccine trials[16,17]. V3 dominance was reduced in the heterotypic DANA 3, 4 and 5, where two to three Env targets were alternated. This allowed more trimer-reactive clones to be selected, but only the triple combination in DANA 5 translated into enhanced neutralization activity of trimer- and V3-reactive clones. This

observation in DANA parallels what has been shown for cross-clade immunization strategies, which favor the induction of a heterologous neutralizing response[18,19]. In particular, trimer-specific DARPins identified from DANA with heterotypic regimens tended to frequently acquire mutations, often extending to the DARPin framework regions. This again proved strikingly similar to bnAbs that target the closed trimer and which across bnAb types harbor a range of unusual genetic features[20,21].

The fact that V3-reactive DARPins were less mutated than trimer-reactive DARPins was highly intriguing. Comparing germline identities (GLI) of variable heavy chains (VH) in HIV-infected individuals with defined bnAb activity we observed the same pattern. Trimer-reactive BCRs in these bnAb inducers were significantly more mutated than V3-reactive BCRs isolated at the same time point. Evidently, in natural infection bnAb affinity maturation is a lengthy process, requiring years of co-evolution of Abs and Env. The DARPin selection, on the other hand, is a concise process and involved in our experimental setup one or few Env variants only. It is therefore particularly intriguing that, despite their genuine differences, the natural antibody response and the DARPin system reacted in a similar way. Both lead to the accumulation of mutations (including framework mutations) to generate binders against the closed trimer, while comparatively lower mutation rates suffice to achieve V3 binding.

By exploring different selection regimens we showed that reduction of V3 dominance benefits the selection of trimer-reactive clones, in agreement with current concepts of HIV-1 vaccine design[17,22,23]. Similar to the evolution of bnAbs, affinity maturation is a critical step to achieve high neutralization activity in DANA (comparison DANA 5 vs 5^{mod})[24-26]. Reducing stringency in selection for affinity can aid in limiting dominant but unwanted high-affinity responses, such as V3-reactivity, but at the same time by enriching low-affinity clones, chances for selecting top neutralizers decrease. The additional polishing of DS-SOSIP trimer preparations used for panning by negative selection (DANA 3mod) appeared to be of limited use. While the trimer-binding fraction of DARPins increased, this measure together with other biopanning adjustments was unable to suppress V3 responses. In addition to V3 specific responses, antibodies directed to the artificial trimer base frequently evolve as off-target responses in vaccinations with highly stabilized, soluble Env trimers[27,28]. Our experimental setup was not designed to pick up trimer base responses as in most DANAs the base was largely covered during panning due to C-terminal immobilization on neutravidin or streptavdin. Altering the immobilization strategy to free off the base could however be used to study effects of trimer-base binders, if of interest.

Additional purifications steps can increase the structural homogeneity of the trimer preparation, the durability of this high purification state, particularly during immunization, may however be limited. Even highly stabilized, trimeric Env proteins may disassemble in vivo[10,11]. As demonstrated by the varying degree of V3 dominance in the different DANA screens, a key feature of DANA is its ability to differentiate between trimer preparations that remain conformationally stable and limit V3 exposure during the selection process and those that do not. Highly stabilized trimer constructs (SOSIP.v4 and SOSIP.v7; DANA 6-9), some of which are in clinical testing (Supplementary Table 1), were close to inert in DANA, yielding mostly highly mutated clones and almost none that were neutralizing. This reflects mouse immunogenicity data of differentially stabilized ConC trimers used in DANA 6 and 8[7]. Mice generated non-neutralizing antibodies to numerous immunodominant regions including V3 but, although these responses were dampened in the conformationally most stable version ConCv5 no neutralizing activity was achieved [7].

Of note, analogous DARPin screens we conducted using Env subunits and low shielded trimers as panning targets, yielded a wide range of neutralizing DARPins[12,13]. It is therefore reasonable to conclude that the protein surface accessible for DARPin binding is limited on highly stabilized trimers. This may allow the DARPin system to initially select only low-affinity clones that require extensive mutation to achieve detectable binding in subsequent rounds of selection.

Overall, our results underscore the ability of DANA to assess epitope accessibility and immunodominant features of candidate Env immunogens and thus confirm the biophysical basis of these processes, as similar trends are seen with DARPins and antibodies, two completely unrelated types of binding molecules.

References to Supplementary Discussion

1 Phanuphak, N. & Gulick, R. M. HIV treatment and prevention 2019: current standards of care. *Curr Opin HIV AIDS* **15**, 4-12, doi:10.1097/COH.0000000000000588 (2020).
2 Mabvakure, B. M. *et al.* Advancing HIV vaccine research with low-cost high-performance computing infrastructure: An alternative approach for resource-limited settings. *Bioinform Biol Insights* **13**, 1177932219882347, doi:10.1177/1177932219882347 (2019).
3 Medina-Ramirez, M., Sanders, R. W. & Sattentau, Q. J. Stabilized HIV-1 envelope glycoprotein trimers for vaccine use. *Curr Opin HIV AIDS* **12**, 241-249, doi:10.1097/COH.0000000000000363 (2017).
4 Torrents de la Pena, A. & Sanders, R. W. Stabilizing HIV-1 envelope glycoprotein trimers to induce neutralizing antibodies. *Retrovirology* **15**, 63, doi:10.1186/s12977-018-0445-y (2018).
5 de Taeye, S. W. *et al.* Stabilization of the gp120 V3 loop through hydrophobic interactions reduces the immunodominant V3-directed non-neutralizing response to HIV-1 envelope trimers. *J Biol Chem* **293**, 1688-1701, doi:10.1074/jbc.RA117.000709 (2018).

6 Torrents de la Pena, A. *et al.* Improving the immunogenicity of native-like HIV-1 envelope trimers by hyperstabilization. *Cell Rep* **20**, 1805-1817, doi:10.1016/j.celrep.2017.07.077 (2017).
7 Hauser, A. *et al.* Stepwise conformational stabilization of a HIV-1 clade C consensus envelope trimer immunogen impacts the profile of vaccine-induced antibody responses. *Vaccines (Basel)* **9**, doi:10.3390/vaccines9070750 (2021).
8 Kwon, Y. D. *et al.* Crystal structure, conformational fixation and entry-related interactions of mature ligand-free HIV-1 Env. *Nat Struct Mol Biol* **22**, 522-531, doi:10.1038/nsmb.3051 (2015).
9 Sanders, R. W. *et al.* A next-generation cleaved, soluble HIV-1 Env trimer, BG505 SOSIP.664 gp140, expresses multiple epitopes for broadly neutralizing but not non-neutralizing antibodies. *PLoS Pathog* **9**, e1003618, doi:10.1371/journal.ppat.1003618 (2013).
10 Antanasijevic, A. *et al.* Structural and functional evaluation of de novo-designed, two-component nanoparticle carriers for HIV Env trimer immunogens. *PLoS Pathog* **16**, e1008665, doi:10.1371/journal.ppat.1008665 (2020).
11 Turner, H. L. *et al.* Disassembly of HIV envelope glycoprotein trimer immunogens is driven by antibodies elicited via immunization. *Sci Adv* **7**, doi:10.1126/sciadv.abh2791 (2021).
12 Friedrich, N. *et al.* Distinct conformations of the HIV-1 V3 loop crown are targetable for broad neutralization. *Nat Commun* **12**, 6705, doi:10.1038/s41467-021-27075-0 (2021).
13 Glögl, M. *et al.* Trapping the HIV-1 V3 loop in a helical conformation enables broad neutralization. *Nat Struct Mol Biol*, doi:10.1038/s41594-023-01062-z (2023).
14 Mann, A. *et al.* Conformation-dependent recognition of HIV gp120 by designed ankyrin repeat proteins provides access to novel HIV entry inhibitors. *J Virol* **87**, 5868-5881, doi:10.1128/JVI.00152-13 (2013).
15 Plückthun, A. Designed ankyrin repeat proteins (DARPins): binding proteins for research, diagnostics, and therapy. *Annu Rev Pharmacol Toxicol* **55**, 489-511, doi:10.1146/annurev-pharmtox-010611-134654 (2015).
16 Sanders, R. W. *et al.* HIV-1 VACCINES. HIV-1 neutralizing antibodies induced by native-like envelope trimers. *Science* **349**, aac4223, doi:10.1126/science.aac4223 (2015).
17 de Taeye, S. W. *et al.* Immunogenicity of stabilized HIV-1 envelope trimers with reduced exposure of non-neutralizing epitopes. *Cell* **163**, 1702-1715, doi:10.1016/j.cell.2015.11.056 (2015).
18 Heydarchi, B. *et al.* Broad and ultra-potent cross-clade neutralization of HIV-1 by a vaccine-induced CD4 binding site bovine antibody. *Cell Rep Med* **3**, 100635, doi:10.1016/j.xcrm.2022.100635 (2022).
19 Dubrovskaya, V. *et al.* Vaccination with glycan-modified HIV NFL envelope trimer-liposomes elicits broadly neutralizing antibodies to multiple sites of vulnerability. *Immunity* **51**, 915-929 e917, doi:10.1016/j.immuni.2019.10.008 (2019).
20 Roskin, K. M. *et al.* Aberrant B cell repertoire selection associated with HIV neutralizing antibody breadth. *Nat Immunol* **21**, 199-209, doi:10.1038/s41590-019-0581-0 (2020).
21 Burton, D. R. & Hangartner, L. Broadly neutralizing antibodies to HIV and their role in vaccine design. *Annu Rev Immunol* **34**, 635-659, doi:10.1146/annurev-immunol-041015-055515 (2016).
22 Sliepen, K. *et al.* Structure and immunogenicity of a stabilized HIV-1 envelope trimer based on a group-M consensus sequence. *Nat Commun* **10**, 2355, doi:10.1038/s41467-019-10262-5 (2019).
23 Havenar-Daughton, C., Lee, J. H. & Crotty, S. Tfh cells and HIV bnAbs, an immunodominance model of the HIV neutralizing antibody generation problem. *Immunol Rev* **275**, 49-61, doi:10.1111/imr.12512 (2017).
24 Bonsignori, M. *et al.* Staged induction of HIV-1 glycan-dependent broadly neutralizing antibodies. *Sci Transl Med* **9**, doi:10.1126/scitranslmed.aai7514 (2017).
25 Bonsignori, M. *et al.* Antibody-virus co-evolution in HIV infection: paths for HIV vaccine development. *Immunol Rev* **275**, 145-160, doi:10.1111/imr.12509 (2017).
26 Bonsignori, M. *et al.* Maturation pathway from germline to broad HIV-1 neutralizer of a CD4-mimic antibody. *Cell* **165**, 449-463, doi:10.1016/j.cell.2016.02.022 (2016).
27 Bianchi, M. *et al.* Electron-Microscopy-based epitope mapping defines specificities of polyclonal antibodies elicited during HIV-1 BG505 envelope trimer immunization. *Immunity* **49**, 288-300 e288, doi:10.1016/j.immuni.2018.07.009 (2018).
28 Nogal, B. *et al.* Mapping polyclonal antibody responses in non-human primates vaccinated with HIV env trimer subunit vaccines. *Cell Rep* **30**, 3755-3765 e3757, doi:10.1016/j.celrep.2020.02.061 (2020).

Supplementary Figures

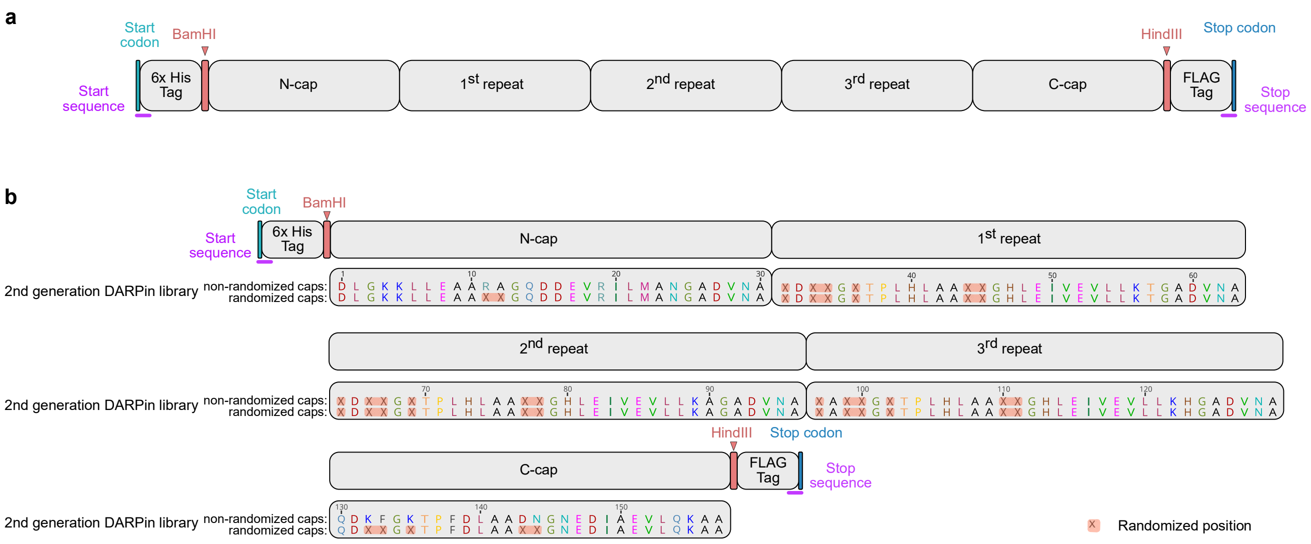

Supplementary Figure 1: Schematic of the Open Reading Frame (ORF) of an N3C DARPin

a The ORF of an N3C DARPin as used in this study comprises, from N- to C-terminus, a His Tag, the N-terminal capping repeats, three internal repeats, the C-terminal capping repeat and a FLAG tag. Start and stop codons are indicated in blue. BamHI and HindIII cloning sites for the DARPin-pool are indicated in red. Sequence stretches (termed start sequence and stop sequence) containing the conserved start or stop codons and comprising 15 nucleotides that were used to validate the DARPin ORF are indicated in violet. **b** DARPin consensus sequences with randomized and non-randomized caps as included in the 2nd gen DARPin library used as starting library in the present study are shown. Randomized positions are marked in red.

		BG505 SOSIP T332N	BG505 DS-SOSIP T332N	30355 SOSIP	30355 DS-SOSIP	sC23v4-KIKO	ConCv5-KIKO	AMC011 SOSIP.v4	ConM SOSIP.v7	ZM197M SOSIP.v4	16055 SOSIP
CD4bs	VRC01	0.50	0.56	6.07	3.85	0.18	0.07	6.24	0.38	0.61	0.02
	PGV04	0.66	0.71	6.64	5.90	0.09	0.07	7.86	0.49	1.83	1.36
	3BNC117	0.46	0.34	1.59	1.35	0.15	0.11	1.78	0.57	1.02	0.74
	b12	14.61	20.00	20.00	20.00	20.00	20.00	20.00	0.54	0.03	0.02
V1V2	PG16	0.67	5.06	20.00	20.00	20.00	3.85	20.00	0.23	20.00	0.02
	PGDM1400	0.34	0.84	17.37	20.00	8.03	2.41	9.59	0.13	14.88	0.70
	PGT145	0.02	0.91	0.07	0.15	0.43	0.48	0.11	0.06	11.08	0.03
V3 glycan	PGT121	0.41	0.45	12.28	20.00	0.54	0.18	0.19	0.08	1.18	0.02
	PGT 128	0.11	0.12	0.24	0.25	0.09	0.10	0.10	0.08	0.43	0.28
Interface	PGT 151	0.21	0.21	20.00	20.00	0.56	2.32	0.36	0.19	20.00	0.82
MPER	10E8	20.00	20.00	20.00	20.00	20.00	20.00	20.00	20.00	20.00	20.00
V3 crown	1-79	0.27	0.14	0.15	0.34	1.11	20.00	1.14	4.06	14.15	0.01
FP	VRC34	0.12	0.14	0.16	0.21	0.34	0.17	0.06	0.12	15.18	0.47
CD4i	17b	20.00	20.00	4.34	20.00	20.00	20.00	20.00	20.00	0.38	2.63
Glycan	2G12	0.02	0.02	0.04	0.07	0.02	0.05	0.01	0.05	2.02	0.20

EC50 (µg/ml): 0.0001, 0.01, 0.1, 1, 10, 20, >20

Supplementary Figure 2: Antigenic characterization of panning trimers

Trimer preparations used for panning were characterized for binding by bnAbs and non-neutralizing Abs (non-nAbs) in a Luminex bead-based immunoassay. For 16055 SOSIP antigenic characterization was performed by ELISA due to difficulties in detection with flow-based Luminex technology. Half maximal binding concentrations (EC50, in µg/ml) shown are geometric means of three independent experiments. Trimer preparations were generated by different research groups (Trkola, Sanders/van Gils and Wagner) and purified by state-of-the-art protocols by affinity and size-exclusion chromatography to isolate the trimer fraction. In case of ConM-SOSIP.v7, ZM197M-SOSIP.v4 and AMC011-SOSIP.v4 purification involved positive selection performed with the quaternary structure specific bnAb PGT145 to enrich the preparation for closed conformation trimers. Depending on the degree of stabilization and purification procedures, Env trimers may contain some partially open trimer proteins. Based on mAb 17b and b12 binding which require considerable opening of the trimer to access their epitopes[51] trimers 16055 SOSIP and ZM197M SOSIP.v4 were ranked as partially open. A modest V3 exposure on stabilized Env trimers is common[3,43,50] and was also detected for most trimers included.

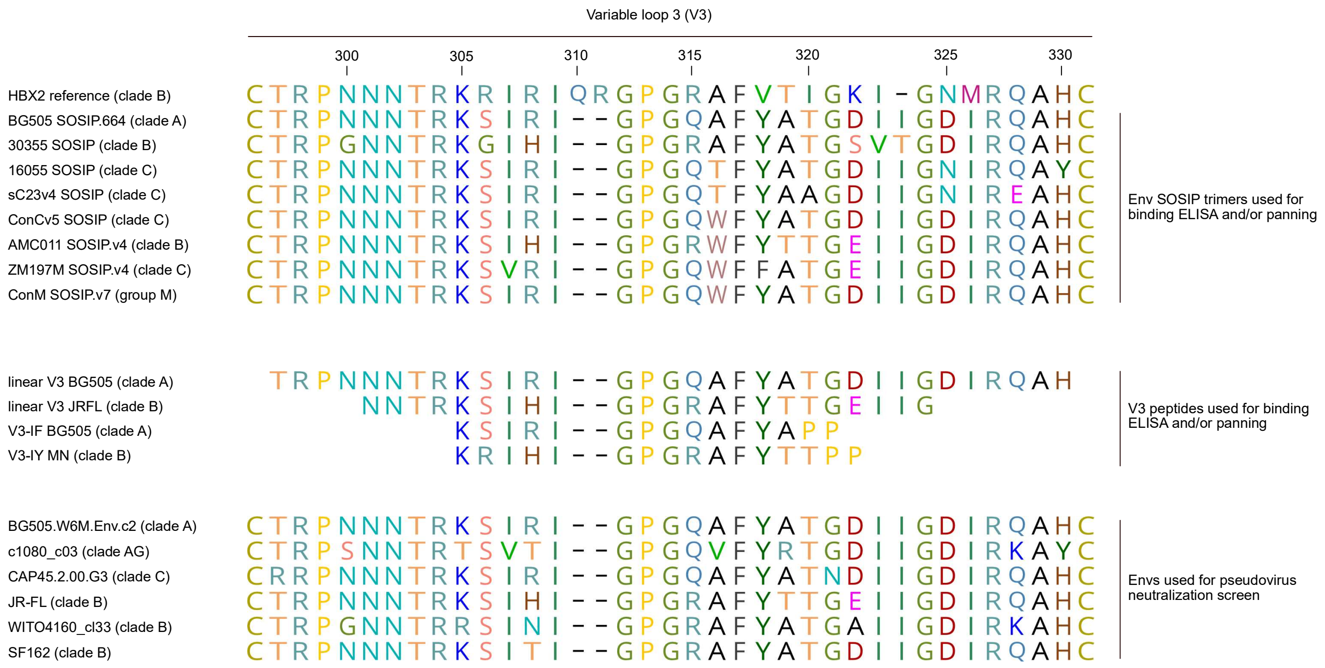

Supplementary Figure 3: Alignment of V3 loop sequences

Amino acid alignment of V3 loop sequences of recombinant stabilized Env trimers (top), peptides (middle) and pseudovirus Envs (bottom) used in this study.

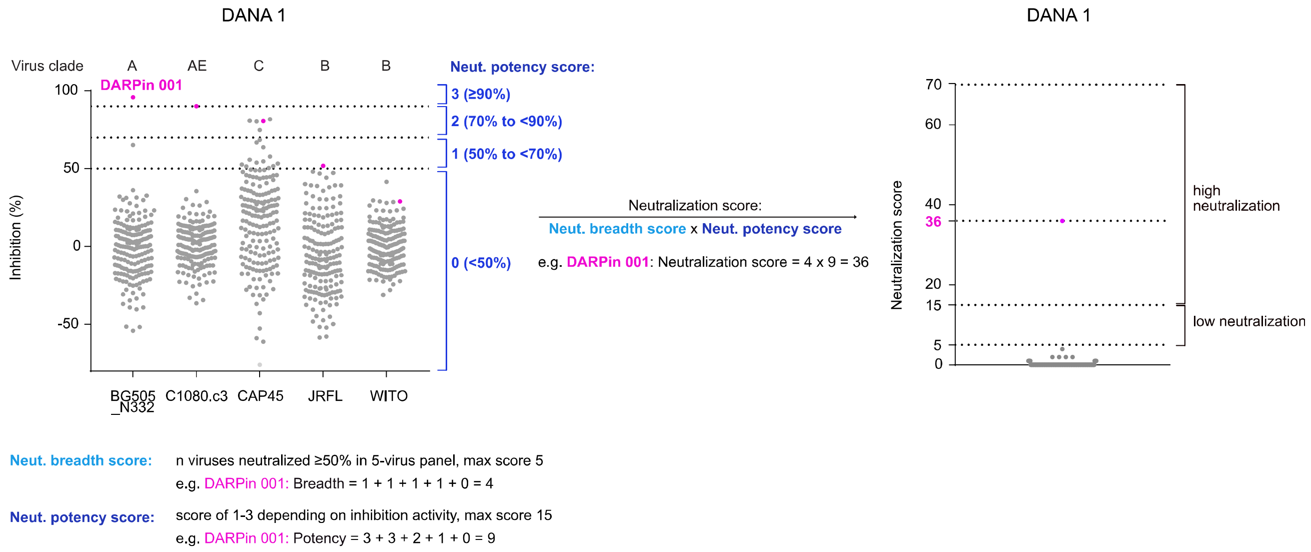

Supplementary Figure 4: Neutralization screening and scoring for DANA 1

Results of the neutralization screen of DANA 1 using Envs of five genetically divergent HIV-1 strains in a TZM-bl-based pseudovirus-neutralization assay are shown to illustrate neutralization scoring. A neutralization potency score of 1 to 3 is assigned based on inhibition of each pseudovirus (dark blue), a neutralization breadth score of 1 is assigned for each virus inhibited ≥50% (light blue). The product of neutralization potency and breadth results in the final neutralization score. In this study, we consider a score of 5-14 as low neutralization and ≥15 as high neutralization. A highly neutralizing clone (DARPin 001) is marked in pink as an example.

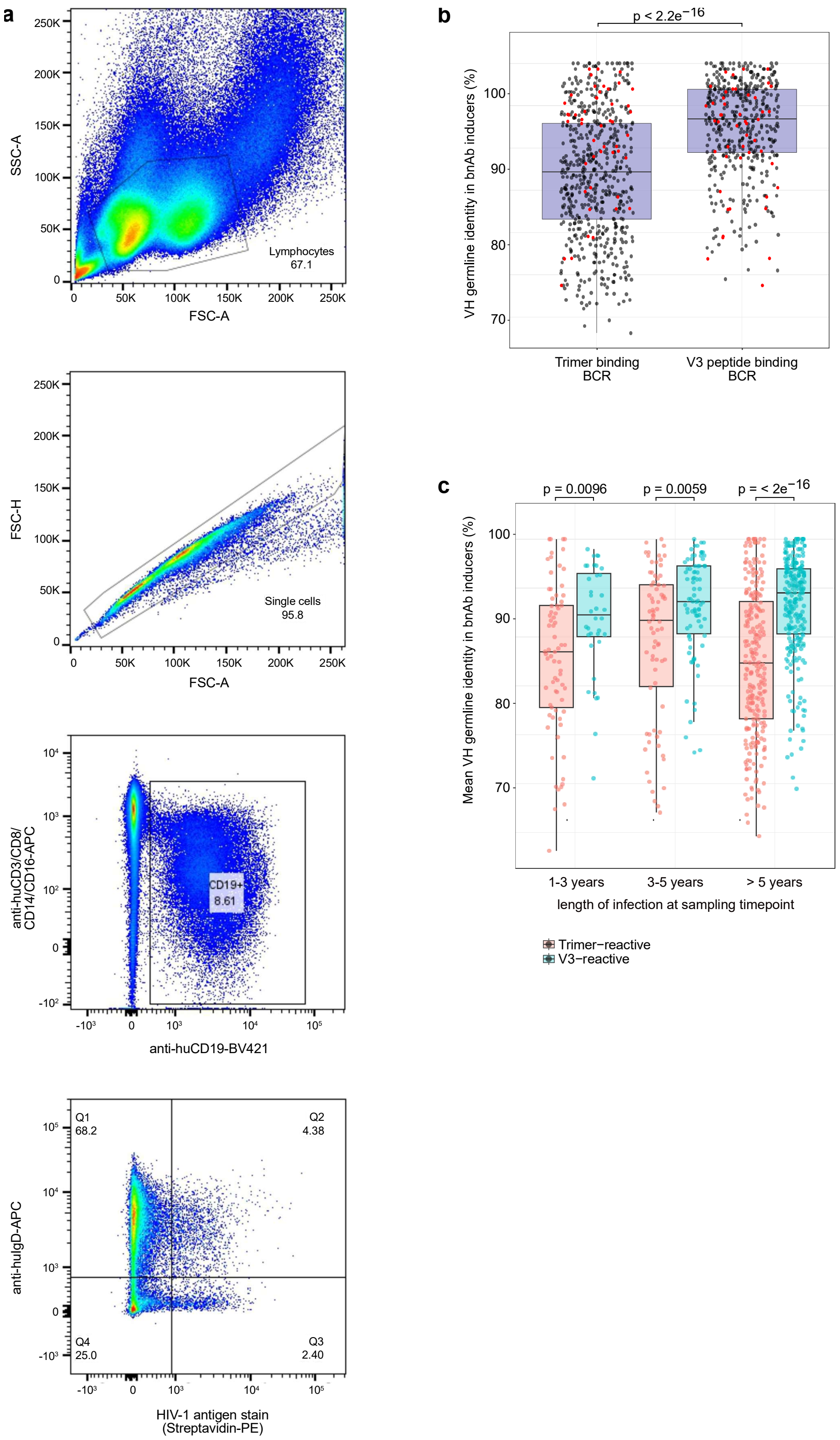

Supplementary Figure 5: Target-specific analysis of variable heavy (VH) chain germline identities in bnAb inducers

Supplementary Figure 5: Target-specific analysis of variable heavy (VH) chain germline identities in bnAb inducers

Analysis of VH chain germline identities (GLI) among 80,963 B cell receptor (BCR) sequences derived from 19 HIV-1 bnAb inducers (total 21 PBMC samples) with target specificity attributed by Libra-seq. Trimer-binding BCR: binds at least one of three trimers (AMC011_SOSIP.v4, ConM_SOSIP.v7, DU422_SOSIP.v4). V3-binding BCR: binds at least one of two V3 peptides (BG505 or JRFL). Boxplots indicate median (middle line), upper and lower quartiles (box limits) and 1.5x interquartile ranges (whiskers). GLIs were compared by two-sided Wilcoxon rank-sum test. **a** Gating strategy (top to bottom) to enrich CD19+/IgD- B cells from PBMCs for BCR sequencing by 10x technology. **b** Comparison of VH chain GLIs of individual trimer reactive (n=605) and V3 peptide-reactive (n=474) BCRs. Trimer- and V3-binding double positive BCRs (n=48) are highlighted. **c** Comparison of mean VH germline identity (GLI) of trimer reactive (n=444) and V3 peptide-reactive (n=431) BCR clonotypes. Trimer- and V3-binding double positive BCRs were excluded from the analysis. Depicted are the same data as in Figure 2a separated by length of infection at the sampling timepoint (1 to 3 years, 3 to 5 years, more than 5 years) as determined in the Swiss 4.5 K study[7].

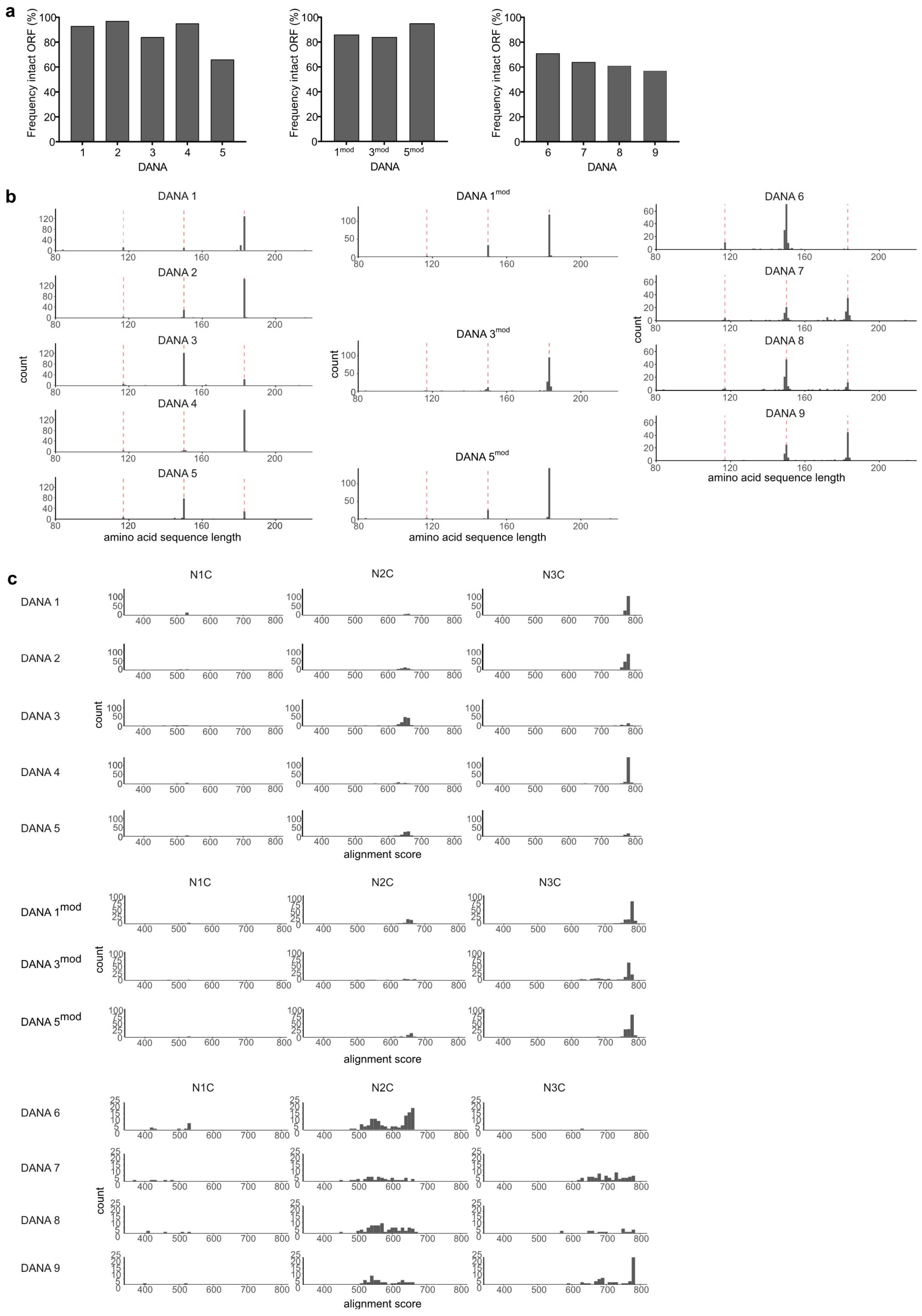

Supplementary Figure 6: Sequencing analysis

Supplementary Figure 6: Sequence analysis

a Frequency of clones with valid ORF. **b** Length distribution of DARPins for each DANA. Red dotted lines indicate length for N1C (117 amino acids), N2C (150 amino acids) and N3C (183 amino acids) DARPins**. c** DARPin clones of each DANA were aligned to the respective consensus sequence of the same DARPin type (N1C, N2C, N3C) in a pairwise alignment and alignment scores were calculated. The higher the alignment score, the closer the sequence is to the consensus framework sequence. Randomized DARPin positions are not considered in this assessment.

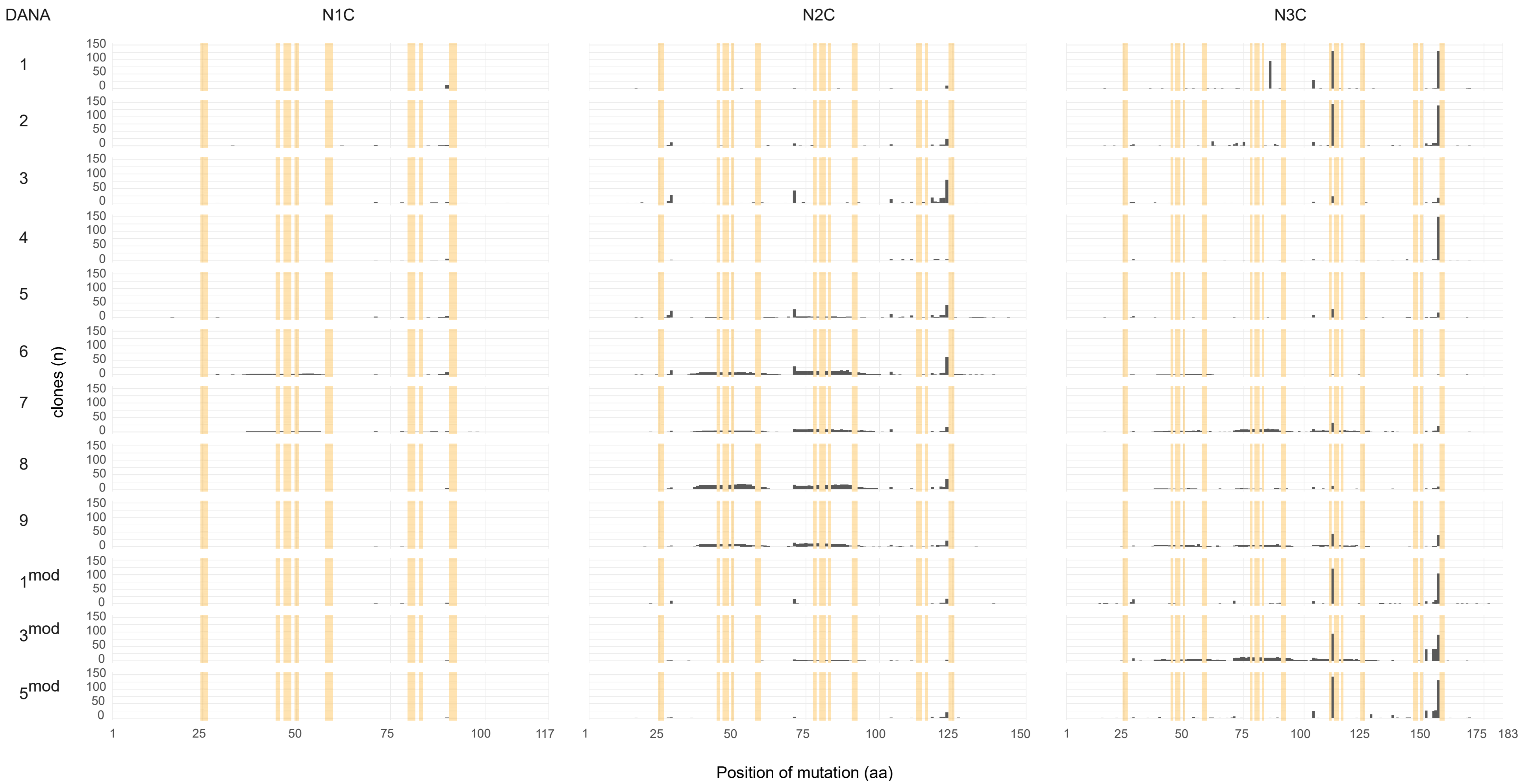

Supplementary Figure 7: Analysis of position-specific substitutions within the DARPin framework

DARPin clones of each DANA were aligned to the respective consensus framework sequence of the same DARPin type (N1C, N2C, N3C) in a pairwise alignment. Substitutions of framework residues in comparison to the consensus sequence were counted in a position-specific manner. Randomized residues are marked by yellow boxes.

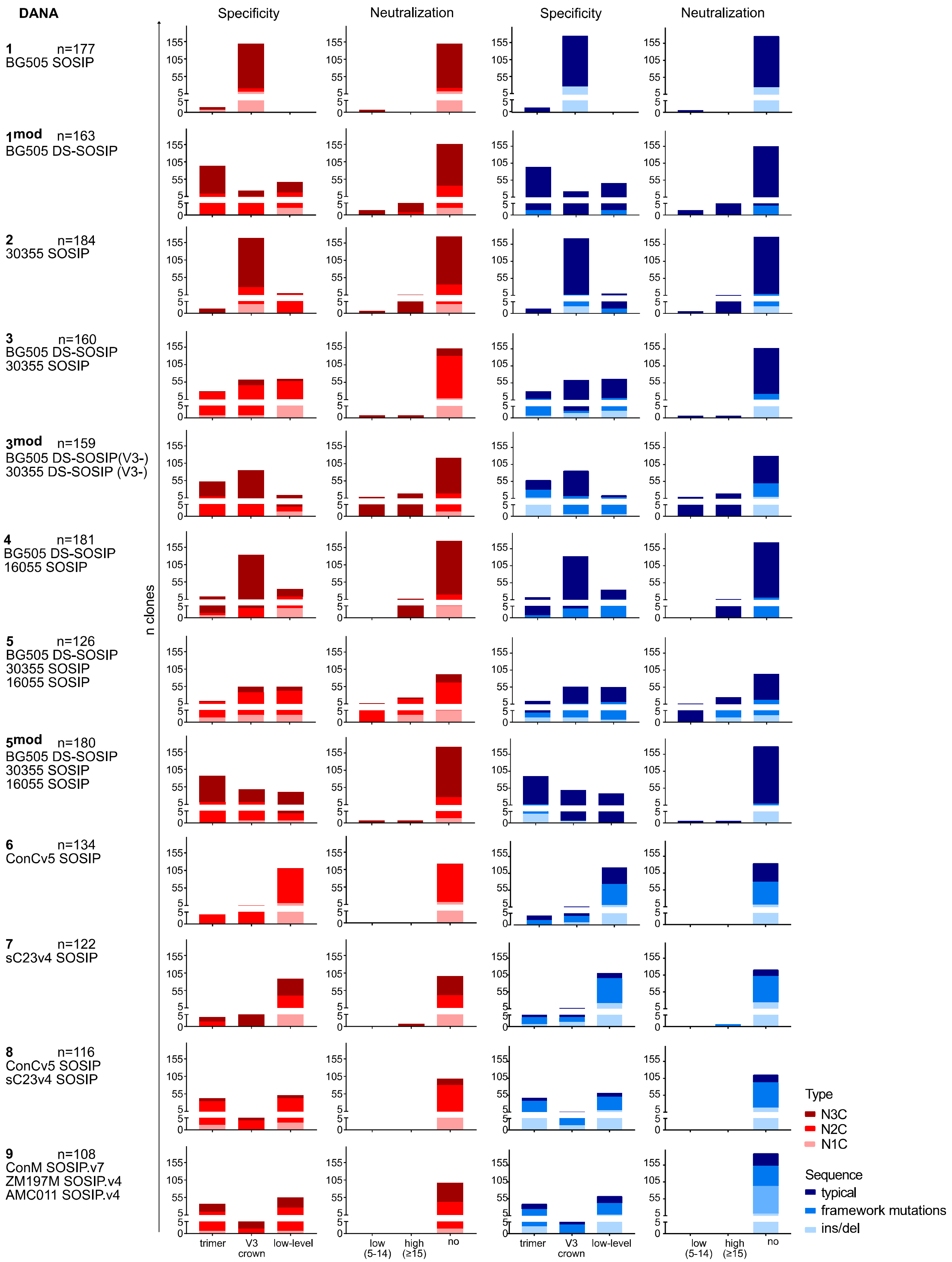

Supplementary Figure 8: Binding properties and neutralization capacities associated with DARPin type and sequence integrity

Distribution of DARPin types and sequence integrity for trimer, V3 crown and low-level binders and no, low and high neutralizers.

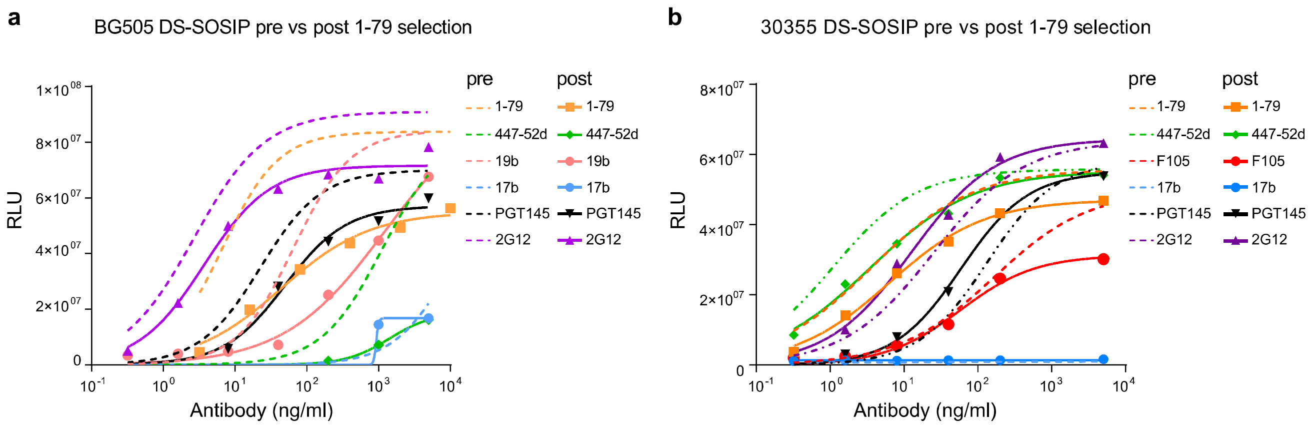

Supplementary Figure 9: Depletion of trimers with exposed V3-loop

Antigenic characterization of 30355 DS-SOSIP and BG505 DS-SOSIP preparations used for DARPin panning. ELISA with HIV mAbs targeting diverse epitopes pre- and post-depletion purification of the trimer preparations with the V3-loop mAb 1-79. Relative light units (RLU) are depicted.

a

Binding									
clone	DANA	ELISA screen	NA	BG505 SOSIP	BG505 DS-SOSIP	BG505 SOSIPΔV1/V2	BG505 SOSIPΔV3	full length V3 (BG505)	V3-IF (BG505)
105.1 A01	5	low-level binder	-	-	n.d.	++	-	-	-
105.1 C01	5	low-level binder	-	+	n.d.	++	-	-	-
105.1 C12	5	low-level binder	-	-	n.d.	++	-	-	-
105.1 G11	5	low-level binder	-	+	+	+	++	-	-
209.1 G08	3mod	trimer	-	++	-	++++	-	-	-
209.2 B11	3mod	trimer	-	++	++	++++	++	-	-
209.2 D02	3mod	trimer	-	++	-	++++	-	-	-
209.2 E03	3mod	trimer	-	+++	-	++++	-	-	-
209.2 E08	3mod	trimer	-	++	-	++++	-	-	-
103.2 G07	5mod	trimer	-	++++	++++	+++	++++	-	-

EC50 [nM]	
-	> 10000
+	10000-1000
++	999-100
+++	99-10
++++	< 10

b

Competition (on BG505 SOSIPΔV1/V2)					
clone	bnD.2	bnD.8	VRC01	F425-B4e8	17b
105.1 A01	+++	+++	-	+	+
105.1 C01	+	-	-	+	-
105.1 C12	++	++	-	+	-
105.1 G11	-	+	-	+	-
209.1 G08	-	+++	-	+	++
209.2 B11	-	+++	-	+	++
209.2 D02	+	+++	-	++	++
209.2 E03	-	+++	-	+	++
209.2 E08	-	+++	-	++	++
103.2 G07	-	-	-	-	-

-	0-39 %
+	40-59 %
++	60-79 %
+++	80-100 %

Supplementary Figure 10: Epitope mapping of several DARPins with virus neutralizing activity

a ELISA-binding of selected DARPins to different targets is indicated by half maximal binding concentrations (EC50, in nM) of single measurements. Unspecific background binding was assessed against neutravidin (NA). **b** Competition Binding ELISA of selected DARPins was performed with BG505 SOSIPΔV1/V2 as target. The indicated competitors as well as DARPin binders were used at binding saturating concentrations. Levels of competition are shown relative to the signal obtained without competitor. All competition assays were performed in duplicates.

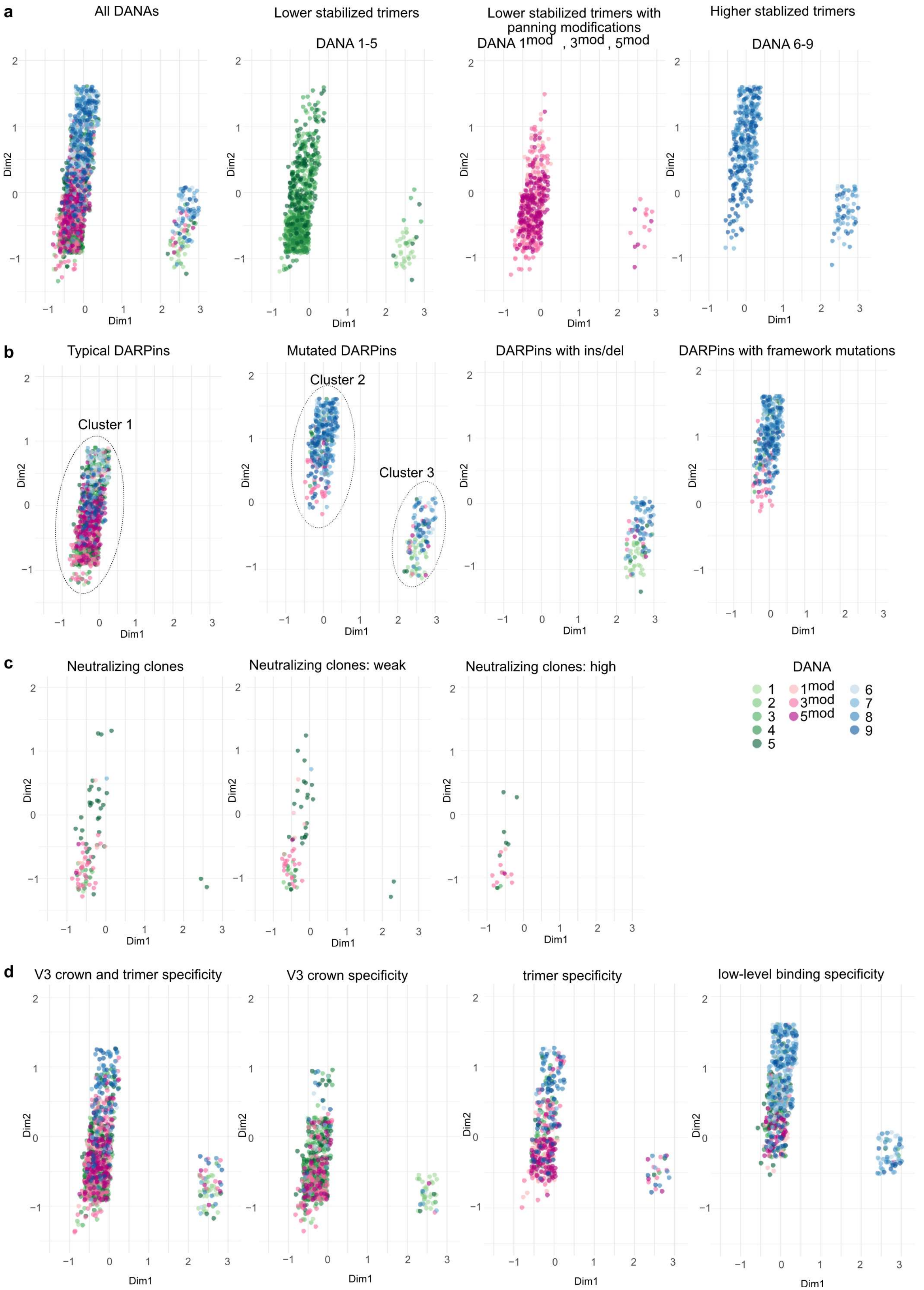

Supplementary Figure 11: Multiple Correspondence Analysis

Supplementary Figure 11: Multiple Correspondence Analysis

A two-dimensional representation of a Multiple Correspondence Analysis (MCA) of 1,810 DARPin clones from all 12 DANA based on binding (V3 crown, trimer, low-level), neutralization (high, low, no), DARPin type (N1C, N2C, N3C, non-classifiable due to insertions or deletions) and DARPin sequence (typical, ins/del, framework mutations). **a** MCA of all DANA, DANA for lower stabilized trimers (DANA 1-5), DANA with modifications for lower stabilized trimers (DANAs 1^{mod}, 3^{mod}, $5^{mod)}$ and DANA 6-9 with higher stabilized trimer. **b** MCA for typical DARPins, total mutated DARPins and specifically DARPins with insertions or deletions and DARPins with framework mutations. **c** MCA for total, weakly and highly neutralizing clones. **d** MCA for total, V3, trimer and low-level binders.

Supplementary Tables

Supplementary Table 1: Env targets used for ribosome display

Env Trimers used in DANA 1-9		
**Trimer type**	**Reference**	**Clinical trial number**
BG505 SOSIP	Sanders, Rogier W., et al. "A next-generation cleaved, soluble HIV-1 Env trimer, BG505 SOSIP. 664 gp140, expresses multiple epitopes for broadly neutralizing but not non-neutralizing antibodies." PLoS pathogens 9.9 (2013): e1003618.	NCT04177355<br>NCT03699241
BG505 DS-SOSIP	Do Kwon, Young, et al. "Crystal structure, conformational fixation and entry-related interactions of mature ligand-free HIV-1 Env." Nature structural & molecular biology 22.7 (2015): 522-531.	NCT04985760
30355 SOSIP	see Sanders, 2013, PLoS Pathog for stabilization approach	-
16055 SOSIP	Guenaga, Javier, et al. "Well-ordered trimeric HIV-1 subtype B and C soluble spike mimetics generated by negative selection display native-like properties." PLoS pathogens 11.1 (2015): e1004570.	-
ConCv5 SOSIP	Hauser, Alexandra, et al. "Stepwise conformational stabilization of a HIV-1 clade C consensus envelope trimer immunogen impacts the profile of vaccine-induced antibody responses." *Vaccines* 9.7 (2021): 750.	-
sC23v4 SOSIP	see Hauser, 2021, Vaccines for stabilization approach	-
ConM SOSIP.v7	Sliepen, K., et al. "Structure and immunogenicity of a stabilized HIV-1 envelope trimer based on a group-M consensus sequence. Nat Commun 10: 2355." (2019).	NCT03961438<br>NCT03816137<br>NCT05208125
ZM197M SOSIP.v4	de Taeye, Steven W., et al. "Immunogenicity of stabilized HIV-1 envelope trimers with reduced exposure of non-neutralizing epitopes." *Cell* 163.7 (2015): 1702-1715.	-
AMC011 SOSIP.v4	van Gils, M. J., et al. "An HIV-1 antibody from an elite neutralizer implicates the fusion peptide as a site of vulnerability. Nat Microbiol 2: 16199." (2016)	-

Supplementary Table 2: Overview of panning targets used in ribosome display rounds in DANA 1-9

	Ribosome display							
DANA	Round 1	Round 2	Round 3	Off target	Additional round	Round 4	Prepanning	ribosome display run
1	BG505 SOSIP	BG505 SOSIP	BG505 SOSIP	BG505 SOSIP	BG505 SOSIP	BG505 SOSIP	-	1 (2014)
2	30355 SOSIP	30355 SOSIP	30355 SOSIP	30355 SOSIP	-	30355 SOSIP	-	2 (2016)
3	30355 SOSIP	BG505 DS-SOSIP	30355 SOSIP	30355 SOSIP	-	BG505 DS-SOSIP	-	2 (2016)
4	BG505 DS-SOSIP	16055 SOSIP	BG505 DS-SOSIP	BG505 DS-SOSIP	-	16055 SOSIP	-	2 (2016)
5	BG505 DS-SOSIP	16055 SOSIP	30355 SOSIP	30355 SOSIP	-	BG505 DS-SOSIP	-	2 (2016)
1_{mod}	BG505 DS-SOSIP	BG505 DS-SOSIP	BG505 DS-SOSIP	-	-	BG505 DS-SOSIP	BG505 SOSIP gp140	2 (2016)
3_{mod}	BG505 DS-SOSIP (V3-)	30355 DS-SOSIP (V3-)	BG505 DS-SOSIP (V3-)	-	-	30355 DS-SOSIP (V3-)	V3-IF (BG505)+V3-IY (MN)	3 (2017)
5_{mod}	BG505 DS-SOSIP	16055 SOSIP	30355 SOSIP	-	-	BG505 DS-SOSIP	-	2 (2016)
6	ConCv5 SOSIP	ConCv5 SOSIP	ConCv5 SOSIP	ConCv5 SOSIP	-	ConCv5 SOSIP	V3-IF (BG505)+V3-IY (MN)	3 (2017)
7	sC23v4 SOSIP	sC23v4 SOSIP	sC23v4 SOSIP	sC23v4 SOSIP	-	sC23v4 SOSIP	V3-IF (BG505)+V3-IY (MN)	3 (2017)
8	ConCv5 SOSIP	sC23v4 SOSIP	ConCv5 SOSIP	sC23v4 SOSIP	-	sC23v4 SOSIP	V3-IF (BG505)+V3-IY (MN)	3 (2017)
9	ConM SOSIP.v7	ZM197M SOSIP.v4	AMC011 SOSIP.v4	BG505 DS-SOSIP	-	ConM.v7 SOSIP	V3-IF (BG505)+V3-IY (MN)	3 (2017)

Supplementary Table 3: Neutralization and binding screen

DANA	1	2	3	4	5
ELISA Targets	V3-IF (BG505)<br>BG505 SOSIP<br>BG505 SOSIP dV3	V3-IF (BG505)<br>BG505 SOSIP dV1V2<br>BG505 DS-SOSIP_V1V2-c1080<br>V2C Mimetic<br>1VH8_CAP256SU	V3-IF (BG505)<br>BG505 SOSIP dV1V2<br>BG505 DS-SOSIP<br>JR-FL gp120<br>1VH8_CAP256SU	V3-IF (BG505)<br>BG505 SOSIP dV1V2<br>BG505 DS-SOSIP<br>JR-FL gp120 dV1V2<br>1VH8_CAP256SU	V3-IF (BG505)<br>BG505 SOSIP dV1V2<br>BG505 DS-SOSIP<br>JR-FL gp120<br>1VH8_CAP256SU
Neutralization Assay Virus Panel	BG505__T332N<br>C1080_c03<br>CAP45_2_00_G3<br>JR-FL<br>WITO4160 clone 33	BG505__T332N<br>C1080_c03<br>CAP45_2_00_G3<br>JR-FL<br>WITO4160 clone 33	BG505__T332N<br>C1080_c03<br>CAP45_2_00_G3<br>JR-FL<br>WITO4160 clone 33	BG505__T332N<br>C1080_c03<br>CAP45_2_00_G3<br>JR-FL<br>WITO4160 clone 33	BG505__T332N<br>C1080_c03<br>CAP45_2_00_G3<br>JR-FL<br>WITO4160 clone 33

DANA	1mod	3mod	5mod	6	7	8	9
ELISA Targets	V3-IF (BG505)<br>BG505 SOSIP dV1V2<br>BG505 DS-SOSIP<br>JR-FL gp120 dV1V2<br>1VH8_CAP256SU	V3-IF (BG505)<br>BG505 SOSIP<br>BG505 DS-SOSIP<br>30355 SOSIP<br>30355 DS SOSIP	V3-IF (BG505)<br>BG505 SOSIP dV1V2<br>BG505 DS-SOSIP<br>JR-FL gp120<br>1VH8_CAP256SU	V3-IF (BG505)<br>BG505 SOSIP<br>BG505 SOSIP dV3<br>ConCv5 SOSIP<br>sC23v4 SOSIP	V3-IF (BG505)<br>BG505 SOSIP<br>BG505 SOSIP dV3<br>ConCv5 SOSIP<br>sC23v4 SOSIP	V3-IF (BG505)<br>BG505 SOSIP<br>BG505 SOSIP dV3<br>ConCv5 SOSIP<br>sC23v4 SOSIP<br>AMC001 SOSIP.v4<br>ConM SOSIP.v7<br>ZM197M SOSIP.v4	V3-IF (BG505)<br>BG505 SOSIP<br>BG505 SOSIP dV3<br>ConCv5 SOSIP<br>sC23v4 SOSIP<br>AMC001 SOSIP.4<br>ConM SOSIP.v7<br>ZM197M SOSIP.v4
Neutralization Assay Virus Panel	BG505__T332N<br>C1080_c03<br>CAP45_2_00_G3<br>JR-FL<br>WITO4160 clone 33	BG505__T332N<br>C1080_c03<br>CAP45_2_00_G3<br>JR-FL<br>WITO4160 clone 33	BG505__T332N<br>C1080_c03<br>CAP45_2_00_G3<br>JR-FL<br>WITO4160 clone 33	BG505__T332N<br>C1080_c03<br>CAP45_2_00_G3<br>JR-FL<br>SF162	BG505__T332N<br>C1080_c03<br>CAP45_2_00_G3<br>JR-FL<br>SF162	BG505__T332N<br>C1080_c03<br>CAP45_2_00_G3<br>JR-FL<br>SF162	BG505__T332N<br>C1080_c03<br>CAP45_2_00_G3<br>JR-FL<br>SF162

Supplementary Table 4: Env-pseudovirus panel

Virus	Subtype	Tier	Genbank entry code
BG505_W6M_C2_T332N	A	2	DQ208458
c1080_c03	AE	2	JN944660
CAP45_2_00_G3	C	2	DQ435682
JR-FL	B	2	AY669728
WITO4160 clone33	B	2	AY835451
SF162	B	1A	EU123924

Supplementary Table 5: Antibody source

**Name**	**Epitope**	**Reference**
b12	CD4bs	Barbas III et al. 1992 PNAS. 89(19):9339-43
VRC34	Interface/Fusionpeptide	Kong et al. 2016 Science. 352(6287), 828-833
3BNC117	CD4bs	Scheid et al. 2011 Science 16;333(6049):1633-7
PGV04	CD4bs	Wu et al. 2010 Science. 329(5993):856-61
VRC01	CD4bs	Wu et al. 2010 Science. 329(5993):856-61
17b	CD4i	Thali et al. 1993 J Virol. 67(7):3978-88
2G12	High Mannose Patch	Trkola et al. 1996 J Virol. 70(2):1100-8
PGT151	Interface/Fusionpeptide	Falkowska et al. 2014 Immunity 40(5): 657–668.
10E8	MPER	Huang et al. 2012 Nature. 491(7424):406-12
PGDM1400	V2 Glycan	Sok et al 2014 PNAS 111(49), 17624-17629
PGT145	V2 Glycan	Walker et al. 2011 Nature. 477(7365):466-70
PG16	V2 Glycan	Walker et al. 2009 Science. 326(5950):285-9
PGT128	V3 High Mannose Patch	Walker et al. 2011 Nature. 477(7365):466-70
PGT121	V3 High Mannose Patch	Walker et al. 2011 Nature. 477(7365):466-70
1-79	V3-Crown	Scheid et al. 2009 Nature. 458(7238):636-40
F425-B4e8	V3-Crown	Pantophlet et al. 2007 Virology. 364:441-53,<br>Bell et al. 2008 J Mol Biol. 375:969-78
