# Supplementary material for: Assessing immunogenicity barriers of the HIV-1 envelope trimer
Source: NPJ Vaccines. 2023 Sep 30;8:148. doi: 10.1038/s41541-023-00746-3 (PMC10542815; doi:10.1038/s41541-023-00746-3)
Supplement: Supplementary file 2 — Reporting-summary [file 41541_2023_746_MOESM2_ESM.pdf]

nature portfolio

Corresponding author(s): Alexandra Trkola

Last updated by author(s): Aug 28, 2023

Reporting Summary

Nature Portfolio wishes to improve the reproducibility of the work that we publish. This form provides structure for consistency and transparency in reporting. For further information on Nature Portfolio policies, see our Editorial Policies and the Editorial Policy Checklist.

Statistics

For all statistical analyses, confirm that the following items are present in the figure legend, table legend, main text, or Methods section.

n/a	Confirmed	
☐	☒	The exact sample size (*n*) for each experimental group/condition, given as a discrete number and unit of measurement
☐	☒	A statement on whether measurements were taken from distinct samples or whether the same sample was measured repeatedly
☐	☒	The statistical test(s) used AND whether they are one- or two-sided<br>*Only common tests should be described solely by name; describe more complex techniques in the Methods section.*
☒	☐	A description of all covariates tested
☒	☐	A description of any assumptions or corrections, such as tests of normality and adjustment for multiple comparisons
☒	☐	A full description of the statistical parameters including central tendency (e.g. means) or other basic estimates (e.g. regression coefficient) AND variation (e.g. standard deviation) or associated estimates of uncertainty (e.g. confidence intervals)
☒	☐	For null hypothesis testing, the test statistic (e.g. *F*, *t*, *r*) with confidence intervals, effect sizes, degrees of freedom and *P* value noted<br>*Give P values as exact values whenever suitable.*
☒	☐	For Bayesian analysis, information on the choice of priors and Markov chain Monte Carlo settings
☒	☐	For hierarchical and complex designs, identification of the appropriate level for tests and full reporting of outcomes
☒	☐	Estimates of effect sizes (e.g. Cohen's *d*, Pearson's *r*), indicating how they were calculated

Our web collection on statistics for biologists contains articles on many of the points above.

Software and code

Policy information about availability of computer code

Data collection	-
Data analysis	All data analyses are detailed in the Materials and Methods section. Binding and neutralization data was analysed using GraphPad PRISM7. Sequences were analysed in R (v4.2.1) and visualized in R (v4.2.1) or in GraphPad PRISM7. Complex heatmap and the multiple component analysis were performed and visualized in R (v4.2.0).

For manuscripts utilizing custom algorithms or software that are central to the research but not yet described in published literature, software must be made available to editors and reviewers. We strongly encourage code deposition in a community repository (e.g. GitHub). See the Nature Portfolio guidelines for submitting code & software for further information.

Data

Policy information about availability of data

All manuscripts must include a data availability statement. This statement should provide the following information, where applicable:

- Accession codes, unique identifiers, or web links for publicly available datasets
- A description of any restrictions on data availability
- For clinical datasets or third party data, please ensure that the statement adheres to our policy

Source data for all displayed items is provided as supplementary files. Raw datasets underlying the source data are available from the corresponding author on request.

Research involving human participants, their data, or biological material

Policy information about studies with human participants or human data. See also policy information about sex, gender (identity/presentation), and sexual orientation and race, ethnicity and racism.

Reporting on sex and gender	*Use the terms sex (biological attribute) and gender (shaped by social and cultural circumstances) carefully in order to avoid confusing both terms. Indicate if findings apply to only one sex or gender; describe whether sex and gender were considered in study design; whether sex and/or gender was determined based on self-reporting or assigned and methods used. Provide in the source data disaggregated sex and gender data, where this information has been collected, and if consent has been obtained for sharing of individual-level data; provide overall numbers in this Reporting Summary. Please state if this information has not been collected. Report sex- and gender-based analyses where performed, justify reasons for lack of sex- and gender-based analysis.*
Reporting on race, ethnicity, or other socially relevant groupings	*Please specify the socially constructed or socially relevant categorization variable(s) used in your manuscript and explain why they were used. Please note that such variables should not be used as proxies for other socially constructed/relevant variables (for example, race or ethnicity should not be used as a proxy for socioeconomic status). Provide clear definitions of the relevant terms used, how they were provided (by the participants/respondents, the researchers, or third parties), and the method(s) used to classify people into the different categories (e.g. self-report, census or administrative data, social media data, etc.) Please provide details about how you controlled for confounding variables in your analyses.*
Population characteristics	The SHCS, founded in 1988, is highly representative of the HIV epidemiology in Switzerland as it includes an estimated 53% of all HIV cases diagnosed in Switzerland since the onset of the epidemic, 72% of all patients receiving ART in Switzerland, and 69% of the nationwide registered AIDS cases.
Recruitment	The Swiss HIV Cohort Study (SHCS) is a prospective, nationwide, longitudinal, non-interventional, observational, clinic-based cohort with semi-annual visits and blood collections, enrolling all HIV-infected adults living in Switzerland.
Ethics oversight	The SHCS has been approved by the ethics committee of the participating institutions (Kantonale Ethikkommission Bern, Ethikkommission des Kantons St. Gallen, Comité départemental d'éthique des spécialités médicales et de médicine communautaire et de premier recours, Kantonale Ethikkommission Zürich, Repubblica e Cantone Ticino - Comitato Ethico Cantonale, Commission cantonale d'éthique de la recherche sur l'être humain, Ethikkommission beider Basel for the SHCS and Kantonale Ethikkommission Zürich for the ZPHI) and written informed consent had been obtained from all participants.

Note that full information on the approval of the study protocol must also be provided in the manuscript.

Field-specific reporting

Please select the one below that is the best fit for your research. If you are not sure, read the appropriate sections before making your selection.

☒ Life sciences ☐ Behavioural & social sciences ☐ Ecological, evolutionary & environmental sciences

For a reference copy of the document with all sections, see nature.com/documents/nr-reporting-summary-flat.pdf

Life sciences study design

All studies must disclose on these points even when the disclosure is negative.

Sample size	The number of samples from HIV infected individuals with broadly neutralizing activity is predetermined through the size of the Swiss HIV-1 Cohort (SHCS) and the Zurich Primary HIV Infection Study (ZPHI) cohort as well as selection criteria applied for the Swiss 4.5K Screen (Rusert et al., Nat. Medicine, 2016). The number of samples (n=21) investigated by antigen-specific BCR sequencing methodology was given by the total number of successful stains conducted with the described antigens at the time of completion for this study.
Data exclusions	No data was excluded, except data from DARPin sequences/clones that did not have a valid ORF (as detailed in the Materials and Methods section under "DARPin sequence analysis" and in the explanation of the workflow in Figure 2c.
Replication	In the binding and neutralization screen of DARPins from each DANA no replicates were done due to the large number of samples. For antigenic characterization of Env trimers (Supplementary Figure 2) the geometric mean of three conducted experiments is shown.
Randomization	no randomization
Blinding	Personnel who conducted the previously reported neutralization screen (Rusert et al., 2016) had no information on patient demographics and neutralization activity at the time of analysis.

Reporting for specific materials, systems and methods

We require information from authors about some types of materials, experimental systems and methods used in many studies. Here, indicate whether each material, system or method listed is relevant to your study. If you are not sure if a list item applies to your research, read the appropriate section before selecting a response.

Materials & experimental systems

n/a	Involved in the study
☐	☒ Antibodies
☐	☒ Eukaryotic cell lines
☒	☐ Palaeontology and archaeology
☒	☐ Animals and other organisms
☒	☐ Clinical data
☒	☐ Dual use research of concern
☒	☐ Plants

Methods

n/a	Involved in the study
☒	☐ ChIP-seq
☐	☒ Flow cytometry
☒	☐ MRI-based neuroimaging

Antibodies

Antibodies used

- mouse anti-FLAG® antibody (Sigma Aldrich, clone M2, Cat#F1804);
- alkaline phosphatase-conjugated polyclonal goat anti-mouse IgG (whole molecule) secondary antibody (Sigma Aldrich, Cat#A3562);
- polyclonal goat anti-human IgG (Fc specific) alkaline phosphatase-conjugated antibody (Sigma-Aldrich, Cat#A9544, Lot. 048K4821)
- phycoerythrin (PE)-labeled secondary antibody specific to isotype IgG1 (Southern Biotech, Cat#9054-09, clone HP6001);
- phycoerythrin (PE)-labeled secondary mouse antibody specific for human IgG-Fc (Southern Biotech, Cat#9040-09, clone JDC-10)
- Allophycocyanin (APC)-labeled mouse anti-human IgD antibody (clone IA6-2, Cat#348222) from Biolegend, San Diego, CA, USA
- APC/Cyanine7-labeled mouse anti-human CD16 antibody (clone 3G8, Cat#302018) from Biolegend, San Diego, CA, USA
- APC/Cyanine7-labeled mouse anti-human CD8 antibody (clone SK1, Cat#344714) from Biolegend, San Diego, CA, USA
- APC/Cyanine7-labeled mouse anti-human CD3 antibody (clone SK7, Cat#344818) from Biolegend, San Diego, CA, USA
- APC/Cyanine7-labeled mouse anti-human CD14 antibody (clone HCD14, Cat#325620) from Biolegend, San Diego, CA, USA
- Brilliant Violet 421-labeled mouse anti-human CD19 antibody (clone HIB19, Cat#302234)
- Mouse anti-human CD4 antibody (clone SK3, Cat#344602) from Biolegend, San Diego, CA, USA
- Barcode-labeled murine antibodies against human CD21 (Totalseq-C0181, clone Bu32), CD27 (Totalseq-C0154, clone O323) and CD38 (Totalseq-C0410, clone HB-7) were purchased from Biolegend, San Diego CA.
- Totalseq barcode- and phycoerythrin (PE)-labeled streptavidin (C0951, Cat#405261; C0952, Cat#405263; C0953, Cat#405265; C0954, Cat#405267; C0955, Cat#405269; C0961, Cat#405155; C0962, Cat#405153; C0963, Cat#405299; C0964, Cat#405297; C0965, Cat#405295) from Biolegend, San Diego, CA, USA
- References for all HIV-1 Envelope protein specific antibodies used in this study are detailed in Supplementary Table 5.

Validation

Mouse anti-FLAG® IgG1 antibody (Sigma Aldrich, clone M2, Cat#F1804 and Cat#F3165) was validated by the company for ELISA, Immunblotting, Immunprecipitation, Immunohistochemistry, Immuncytochemistry, and Immunfluorescence-Assays and has been optimized for detection of FLAG-tagged proteins in mammalian, plant and bacterial expression systems. The M2 antibody is not Calcium dependent (unlike clone M1) and is able to recognize the FLAG-tag at the N-terminus, C-terminus and at internal sites.
Brilliant Violet 421-labeled mouse anti-human CD19 antibody (clone HIB19), APC-labeled mouse anti-human IgD antibody (clone IA6-2), mouse anti-human CD4 antibody (clone SK3) as well as APC/Cyanine7-labeled mouse antibodies against human CD3 (clone SK7), human CD8 (clone SK1), human CD14 (clone HCD14) and human CD16 (clone 3G8) were quality control tested by the manufacturer (Biolegend, San Diego, CA, USA) by immunofluorescent staining with flow cytometric analysis. Each lot of Totalseq-C barcode-labeled antibody is quality control tested by the manufacturer (Biolegend, San Diego, CA, USA) by immunofluorescent staining with flow cytometric analysis and the oligomer sequence is confirmed by sequencing.
PE-labeled secondary mouse antibody specific for human IgG-Fc (Southern Biotech, Cat#9040-09, clone JDC-10) was quality tested by ELISA, FLISA and Flow Cytometry by the manufacturer.
All HIV-1 Envelope protein specific antibodies were validated in the references provided in Supplementary Table 5 and their specificity was verified by the use of appropriate background controls in the current study.

Eukaryotic cell lines

Policy information about cell lines and Sex and Gender in Research

Cell line source(s)

HEK 293-T cells were obtained from the American Type Culture Collection and TZM-bl cells through the NIH AIDS Reagent Program. HEK 293T FreestyleTM suspension (293F and Expi293F) cells were purchased from Thermo Fisher.

Authentication

None of the cell lines used were authenticated again after reception from the specified original source.

Mycoplasma contamination

In the Trkola laboratory, cell lines are routinely tested for mycoplasma contamination. No such contamination was detected in the cells used for the present study.

Commonly misidentified lines
(See ICLAC register)

No commonly misidentified cell lines were used in the study.

Flow Cytometry

Plots

Confirm that:

☒ The axis labels state the marker and fluorochrome used (e.g. CD4-FITC).

☒ The axis scales are clearly visible. Include numbers along axes only for bottom left plot of group (a 'group' is an analysis of identical markers).

☒ All plots are contour plots with outliers or pseudocolor plots.

☒ A numerical value for number of cells or percentage (with statistics) is provided.

Methodology

Sample preparation	The following information is taken from the Materials and Methods section under "Preparation of memory B cells for single cell BCR seqencing": Cryo-preserved peripheral blood mononuclear cells (PBMC, approx. 10E7 cells) from previously identified HIV-1 bnAb inducers were obtained from the SHCS biobank. Cells were thawed, washed, and stained in PBS with 2% FCS with fluorescently labelled antibodies for enriching B cells by sorting and TotalseqC barcode-labelled probes and antibodies for LIBRA-seq (Linking B cell receptor to an antigen) profiling. Fluorescently labelled mouse monoclonal antibodies (APC-Cy7 conjugated antibodies against human CD16 (clone 3G8), CD14 (clone HCD14), CD8 (clone SK1), and CD3 (clone SK7) as well as APC conjugated antibody against human IgD (clone IA6-2) and Brilliant Violet 421 conjugated antibody against human CD19 (clone HIB19)) and TotalseqC barcode-labelled antibodies to CD21 (C0181, clone Bu32), CD27 (C0154, clone O323) and CD38 (C0410, clone HB-7) were purchased from Biolegend, San Diego CA. For TotalseqC barcoding of HIV-1 antigens, biotinylated HIV-1 antigens were incubated with TotalseqC streptavidin-PE (Biolegend, San Diego CA) overnight at room temperature according to the manufacturer's instructions and then stored at 4 °C for up to 30 days. TotalseqC barcode labelled antigens included soluble, stabilized HIV-1 Env trimers (AMC011 SOSIP.v4, ConM SOSIP.v7, DU422 SOSIP.v4) and linear V3 peptides (V3_JRFL, V3_BG505) (see Supplementary Fig. 3 for V3 sequences). Fluorescently labelled mAbs and TotalseqC labelled PBMC were incubated for 30 min at room temperature, washed (PBS with 2% FCS) and CD19 positive, IgD negative B cells (comprising memory B cells and plasmablasts) were sorted on a BD FACSCalibur™ System (Beckton Dickinson, USA) (see Supplementary Fig. 5a).
Instrument	BD FACSAria™ III System (Beckton Dickinson, USA)
Software	FlowJo_v10
Cell population abundance	Post-sort fractions were not re-analyzed for purity.
Gating strategy	A representative experiment describing the gating strategy for the enrichment of CD19 positive/IgD negative B cells from PBMCs is provided in Supplementary Fig. 5a.

☒ Tick this box to confirm that a figure exemplifying the gating strategy is provided in the Supplementary Information.
